# Host-Environment Interplay Shapes Fungal Diversity in Mosquitoes

**DOI:** 10.1128/mSphere.00646-21

**Published:** 2021-09-29

**Authors:** Patil Tawidian, Kerri L. Coon, Ari Jumpponen, Lee W. Cohnstaedt, Kristin Michel

**Affiliations:** a Division of Biology, Kansas State University, Manhattan, Kansas, USA; b Department of Bacteriology, University of Wisconsin—Madison, Madison, Wisconsin, USA; c Arthropod-Borne Animal Diseases Research Unit, Center for Grain and Animal Health Research, Manhattan, Kansas, USA; University of Michigan—Ann Arbor

**Keywords:** fungal community, microbiota, *Aedes albopictus*, insect, gut, diversity, mycobiota

## Abstract

Mosquito larvae encounter diverse assemblages of bacteria (i.e., “microbiota”) and fungi in the aquatic environments that they develop in. However, while a number of studies have addressed the diversity and function of microbiota in mosquito life history, relatively little is known about mosquito-fungus interactions outside several key fungal entomopathogens. In this study, we used high-throughput sequencing of internal transcribed spacer 2 (ITS2) metabarcode markers to provide the first simultaneous characterization of the fungal communities in field-collected Aedes albopictus larvae and their associated aquatic environments. Our results reveal unprecedented variation in fungal communities among adjacent but discrete larval breeding habitats. Our results also reveal a distinct fungal community assembly in the mosquito gut versus other tissues, with gut-associated fungal communities being most similar to those present in the environment where larvae feed. Altogether, our results identify the environment as the dominant factor shaping the fungal community associated with mosquito larvae, with no evidence of environmental filtering by the gut. These results also identify mosquito feeding behavior and fungal mode of nutrition as potential drivers of tissue-specific fungal community assembly after environmental acquisition.

**IMPORTANCE** The Asian tiger mosquito, Aedes albopictus, is the dominant mosquito species in the United States and an important vector of arboviruses of major public health concern. One aspect of mosquito control to curb mosquito-borne diseases has been the use of biological control agents such as fungal entomopathogens. Recent studies also demonstrate the impact of mosquito-associated microbial communities on various mosquito traits, including vector competence. However, while much research attention has been dedicated to understanding the diversity and function of mosquito-associated bacterial communities, relatively little is known about mosquito-associated fungal communities. A better understanding of the factors that drive fungal community diversity and assembly in mosquitoes will be essential for future efforts to target mosquito-associated bacteria and fungi for mosquito and mosquito-borne disease control.

## INTRODUCTION

Mosquito larvae and adults continuously encounter diverse microorganisms in their aquatic and terrestrial environments (1–3). These microorganisms include bacteria and fungi, which assemble into bacterial and fungal communities (defined as collections of species occurring together in the same place at the same time [4, 5]) that can be recovered from the gut using culture-dependent and culture-independent methods. The bacterial communities associated with mosquito larvae are environmentally acquired from the larval breeding water through feeding (6–9) and form largely transient associations with mosquito larvae (10, 11). Nevertheless, the mosquito gut bacterial communities play a profound role in the growth and development of larvae as well as adult survival, fecundity, and mosquito-borne transmission of disease-causing pathogens (9, 12–18). In contrast, studies of fungus-mosquito interactions have largely focused on the identification of fungal entomopathogens and their use as bioinsecticides to control mosquito larvae and adults (19–23). Of the 158 fungal species observed in or isolated across mosquito species, nearly two-thirds are entomopathogens (2). These entomopathogenic fungi infect mosquitoes mostly through the cuticle and rarely through ingestion (2). However, not all fungus-mosquito interactions have a negative outcome on mosquitoes. Fungi such as yeasts can be sufficient for mosquito development as a nutritional source and because they induce gut hypoxia, which serves as a cue for larval development (10, 18, 24). Taken together, these studies strongly suggest that fungi have the potential to profoundly impact mosquito biology, yet very few studies have examined the factors shaping fungal communities in larvae and the larval environments where they naturally develop.

Studies that have focused on the characterization of microbial diversity in mosquitoes collectively indicate that bacterial communities can vary substantially across different larval environments and between individuals that co-occur in the same environment, even at small local scales (6, 8, 9). Studies also indicate that the majority of bacteria present in mosquitoes are restricted to the gut (25–27). The few available published studies of fungal communities suggest that environment may also be a dominant factor shaping fungal diversity in mosquitoes (28). However, to date, no study has simultaneously characterized the fungal communities in mosquito larvae and the aquatic environment that they inhabit. In addition, studies thus far have largely focused on the characterization of fungal communities in only either whole adult mosquitoes or their dissected guts (29–32). Fungi also form associations with their mosquito hosts as a function of their mode of nutrition: some taxa enter the mosquito through the body surface (cuticle), whereas others enter via ingestion through the gut. However, no study to date has examined whether fungal communities differ between mosquito host tissues.

The overall goal of this study was to determine the factors that shape fungus-mosquito interactions in larvae of the Asian tiger mosquito (Aedes albopictus), an abundant mosquito species of public health concern because adult females transmit the causative agents of dengue fever, chikungunya, and Zika (33–36). A. albopictus is ubiquitous in urban and periurban areas throughout most of the world, where larvae inhabit diverse natural and man-made containers (37–39) and feed on living and decaying organic matter using diverse filtering, grazing, and shredding behaviors (40, 41). Here, we sampled water and late-stage (L4) *A. albopictus* larvae from several types of man-made container breeding sites on a fine geographic scale. We then used internal transcribed spacer 2 (ITS2) metabarcoding to determine the fungal community composition and diversity in mosquito larvae and their larval breeding water. Using these data, we determined that the aquatic environment is the major driver of mosquito fungal community composition. We also identified additional drivers, including mosquito feeding behavior and fungal mode of nutrition, that contribute to fungal community assembly and diversity in different mosquito tissues.

## RESULTS

### Fungal communities based on ITS2 metabarcoding.

**FIG 1:**
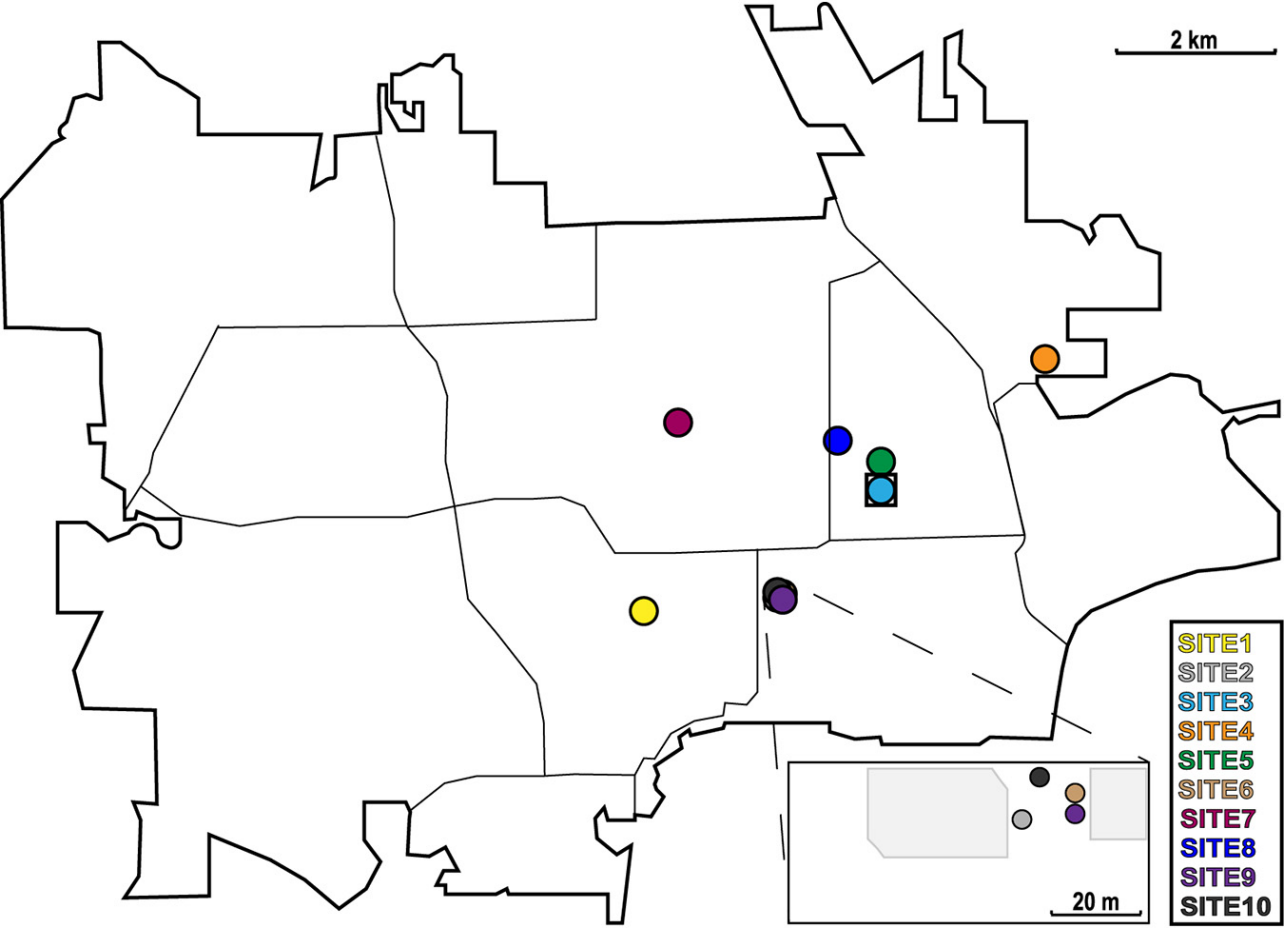
Collection sites for the ITS2 libraries prepared from water and Aedes albopictus mosquito larvae. The location of each collection site in Manhattan, KS, is depicted. Sites 1 and 2 are natural *A. albopictus* breeding sites, while the remaining sites were artificially constructed using plastic mosquito oviposition cups lined with germination paper. The square surrounding site 3 indicates that this site was eliminated from downstream community analyses due to a low number of sequencing reads.

**FIG 2:**
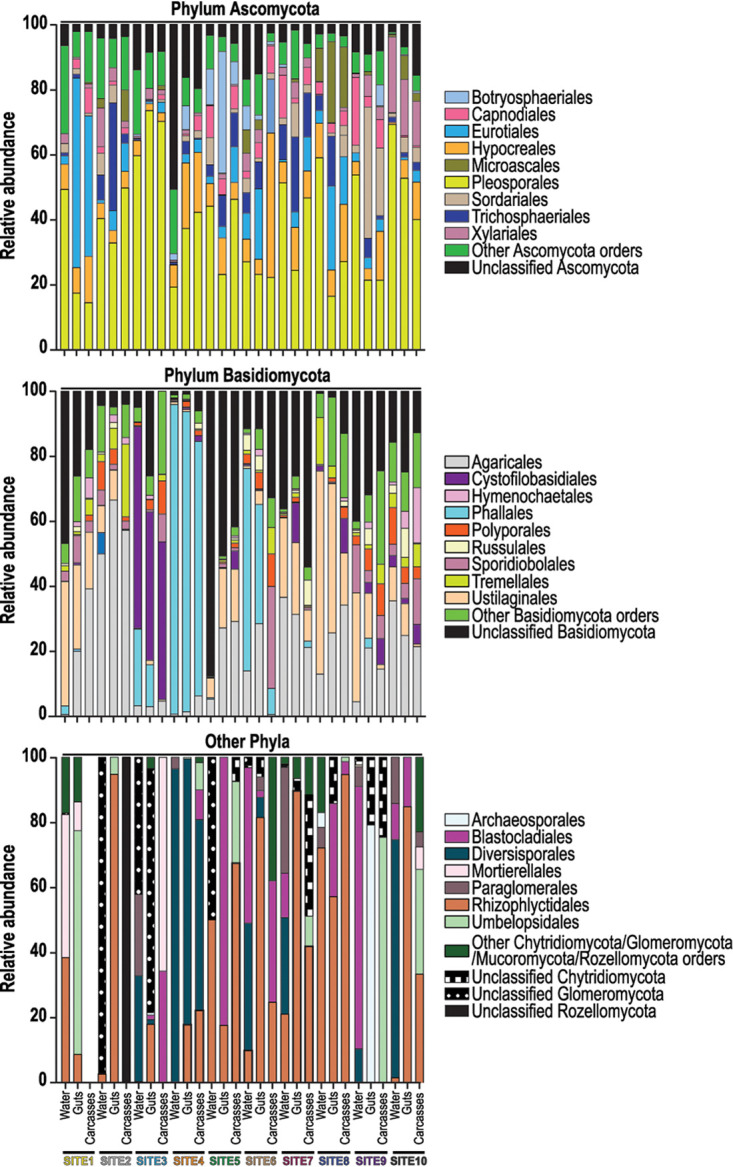
Fungi at the levels of phylum and order in water and mosquito larvae from each collection site. Gut and carcass samples from individual larvae collected from a given site were pooled for the bar graphs presented. For a given phylum, bars present the proportion of sequencing reads assigned to that phylum that were also classified to a specific fungal order. All orders that represented ≥10% of the reads from a given sample are listed in the key; less abundant orders are grouped under the “Other” categories.

We analyzed the fungal communities associated with *A. albopictus* larvae and water sampled from 10 aquatic breeding sites located within an 11.6-km^2^ area in Manhattan, KS (Fig. 1). From each site, we sampled ∼50 ml of water and 10 individual larvae. The larvae were aseptically dissected to produce paired gut and carcass samples prior to sequencing fungal ITS2 metabarcode amplicons on an Illumina MiSeq platform. The final data set consisted of a total of 4,259,124 quality-filtered sequences, assigned to a total of 3,415 operational taxonomic units (OTUs) at a sequence similarity cutoff threshold of 97%. Rarefaction curves saturated or nearly saturated at 5,000 sequences for most samples, indicating that the vast majority of fungal diversity was captured in our sampling (see Fig. S1 in the supplemental material). This is consistent with the high Good’s coverage estimates that we observed across all samples (0.999 ± 0.001 standard deviation). Six fungal phyla were identified across all samples and accounted for 91.1% of the total quality-filtered reads: Ascomycota (59.5%), Basidiomycota (30.8%), Chytridiomycota (0.316%), Glomeromycota (0.057%), Mucoromycota (0.465%), and Rozellomycota (0.019%) (Fig. 2). The remaining 8.86% of reads could not be classified past the kingdom Fungi but were included in all downstream analyses.

10.1128/mSphere.00646-21.2 FIG S1 Rarefaction curves. Graphs show plotted rarefaction data from Illumina sequencing of all water (A and D) and mosquito gut (B and E) and carcass (C and F) libraries prepared for the study using fungal OTUs (top row) and ASVs (bottom row). Rarefaction was performed prior to excluding paired mosquito samples for which we obtained <1,500 sequences. Each site is shown with a different color. In contrast to those observed with the OTU sequencing approach, the rarefaction curves using the ASV approach did not reach saturation, indicating that the fungal communities in the water and mosquito samples were not entirely captured in our sampling process. Download FIG S1, PDF file, 0.2 MB. Copyright © 2021 Tawidian et al. 2021 Tawidian et al. https://creativecommons.org/licenses/by/4.0/ This content is distributed under the terms of the Creative Commons Attribution 4.0 International license.

Within the phylum Ascomycota, OTUs in the fungal orders Pleosporales, Hypocreales, and Eurotiales were shared across all water and mosquito samples and represented ∼38.1%, 10.2%, and 9.5% of the total reads assigned to this phylum, respectively (Fig. 2). Within the phylum Basidiomycota, the majority of reads (∼21.8%) were associated with the order Agaricales, and reads assigned to this order were detected in all of the water and mosquito samples (Fig. 2). In contrast, fungi of the orders Cystofilobasidiales and Phallales were abundant in the water and mosquito gut and carcass samples from only sites 3 and 4 (20.9% and 31.5%, respectively) but rare (1.87% on average) in samples from other sites (Fig. 2). The majority of reads (45.0% on average) within the remaining four phyla belonged to the orders Rhizophlyctidales (phylum Chytridiomycota) and Mortierellales (phylum Mucoromycota) (Fig. 2).

### Local environment is the dominant factor that shapes the fungal community associated with mosquito larvae.

**FIG 3:**
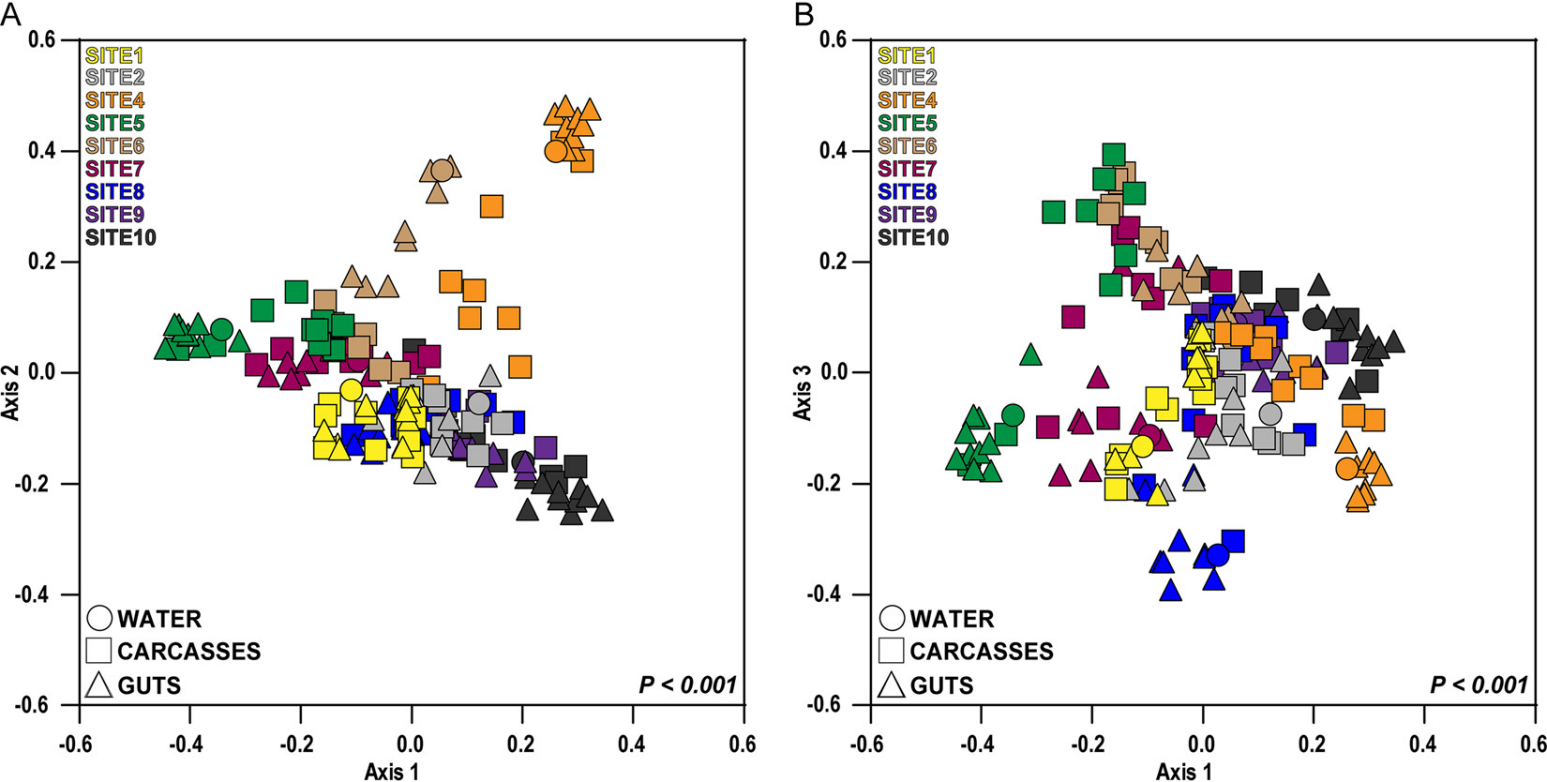
Principal-coordinate analysis based on pairwise Bray-Curtis distances. The keys at the top left of each plot designate collection site by color, while the keys at the bottom left of each plot designate sample type (water versus mosquito gut or carcass) by symbol shape. Samples cluster significantly by collection site (*F* = 7.167; *P < *0.001 [by PERMANOVA]).

To identify whether environment is a major driver of fungal diversity associated with mosquito larvae, we visualized Bray-Curtis dissimilarities among all our water, gut, and carcass samples using principal-coordinate analysis (PCoA) (Fig. 3 and Fig. S2). To determine whether samples were distinct between breeding sites, we analyzed the distance matrices using permutational multivariate analysis of variance (PERMANOVA). The results revealed significant differences in fungal diversity between samples from different sites (Fig. 3 and Fig. S2). We then ran multivariate analysis of variance (MANOVA) on the distance matrices of the first three PCoA vectors to determine whether the fungal communities between breeding sites and/or mosquito tissue types (gut versus carcass) were significantly different. Univariate analyses of variance (ANOVAs) were performed on significant MANOVA factors to examine which axis or axes drove any patterns observed in the PCoA plots. The MANOVA and univariate ANOVAs further indicated that differences along all three PCoA axes were significant for site and the interaction of site and mosquito tissue type, but samples were separated only by tissue along the third axis (Tables 1 and 2 and Table S1).

**TABLE 1:** Contribution of collection sites and mosquito tissues to the fungal community associated with mosquito larvae determined by multivariate analysis of mosquito guts and carcasses (mosquito tissues) across all collection sites

Source of variation	Pillai’s trace	Hypothesis df	Error df	*F*	*P*
Collection sites	2.26	24.0	456	57.8	<0.0001
Mosquito tissues	0.348	3.00	150	26.7	<0.0001
Collection sites: mosquito tissues	1.04	24.0	456	10.0	<0.0001

**TABLE 2:** Contribution of collection sites and mosquito tissues to the fungal community associated with mosquito larvae determined by univariate analyses of PCoA axes

Source of variation in univariate analysis	Sum of squares	Residual sum of squares	df	*F*	*P*
PCoA axes driving variation between mosquito tissues					
Axis 1	0.001	5.55	1.00	0.039	0.844
Axis 2	0.018	4.52	1.00	0.686	0.409
Axis 3	0.616	4.34	1.00	27.8	<0.0001

PCoA axes driving variation between collection sites					
Axis 1	4.38	5.55	8.00	75.4	<0.0001
Axis 2	3.38	4.52	8.00	59.8	<0.0001
Axis 3	1.83	4.34	8.00	14.6	<0.0001

PCoA axes driving interaction between collection sites and tissues					
Axis 1	0.390	1.17	8.00	9.52	<0.0001
Axis 2	0.453	1.12	8.00	12.9	<0.0001
Axis 3	0.591	1.89	8.00	8.60	<0.0001

10.1128/mSphere.00646-21.3 FIG S2 Bray-Curtis dissimilarities within each site. (A) Principal-coordinate analyses based on the pairwise Bray-Curtis distances between mosquito gut and carcass samples collected from a given site. Statistical significance was determined using separate permutational multivariate analyses of variance for each site. (B) Bray-Curtis distances between fungal communities detected in water and either the guts or carcasses of mosquito larvae collected from the same site. Connected lines depict matching mosquito gut and carcass samples for a given collection site. Statistical significance was determined using separate Wilcoxon signed-rank tests for each site. Download FIG S2, PDF file, 0.3 MB. Copyright © 2021 Tawidian et al. 2021 Tawidian et al. https://creativecommons.org/licenses/by/4.0/ This content is distributed under the terms of the Creative Commons Attribution 4.0 International license.

10.1128/mSphere.00646-21.5 TABLE S1 Alpha diversity indices of the larval breeding water and mosquito guts and carcasses across the larval breeding sites. Download Table S1, PDF file, 0.1 MB. Copyright © 2021 Tawidian et al. 2021 Tawidian et al. https://creativecommons.org/licenses/by/4.0/ This content is distributed under the terms of the Creative Commons Attribution 4.0 International license.

### Tissue-specific patterns of fungal community assembly.

**FIG 4:**
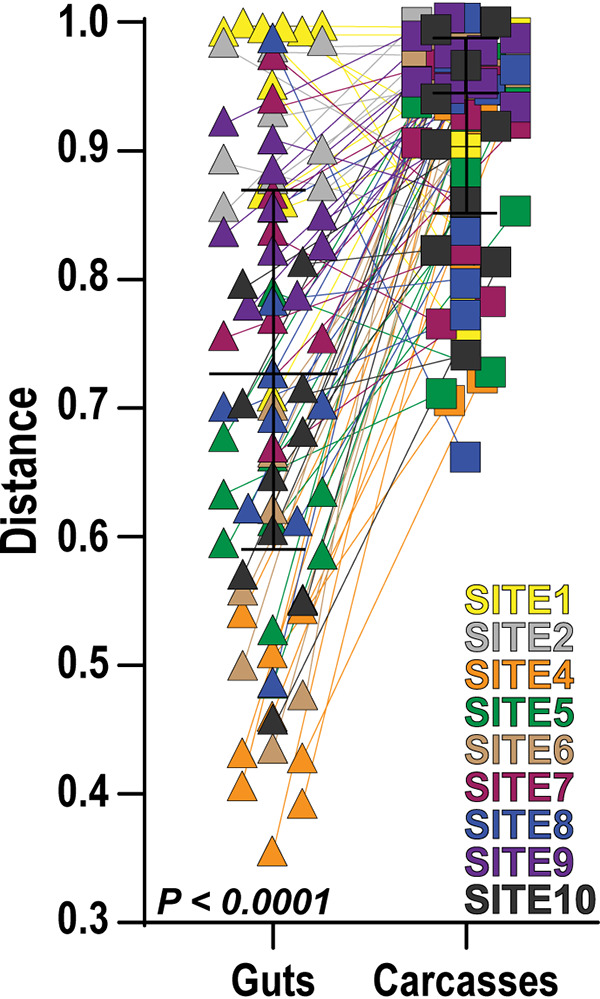
Bray-Curtis distances between fungal communities detected in water and either the guts or carcasses of mosquito larvae collected from the same site. Connected lines depict matching mosquito gut and carcass samples for a given collection site. Statistical significance was determined using a Wilcoxon signed-rank test.

To test whether patterns of fungal community assembly differ between the mosquito gut and other tissues, we next compared the calculated indices for alpha diversity and community composition within all water and individual mosquito gut and carcass samples that we collected. Alpha diversity differed between mosquito tissues (gut versus carcass) as measured by observed (*W* = −3,165; *P* < 0.0001) and extrapolative (*W* = −3,153; *P* < 0.0001) Chao1 OTU richness as well as Shannon’s diversity (*W* = −1,722; *P* < 0.0001). Fungal species richness and diversity were consistently higher in mosquito gut samples than in the corresponding carcass samples (Table S1). Alpha diversity was also generally higher in the water than in the mosquito samples (Table S1). Interestingly, the pairwise Bray-Curtis dissimilarities were higher between water and carcass samples than between water and gut samples (Fig. 4; Fig. S2), indicating that the water and mosquito gut fungal communities were on average more similar than those of the water and mosquito carcasses. We also analyzed the distance matrices between individual gut and carcass samples by site using PERMANOVA. The two mosquito tissues differed in the majority of sites, suggesting distinct fungal communities in mosquito guts and carcasses (Fig. S3).

10.1128/mSphere.00646-21.4 FIG S3 ASV analysis parallels the OTU analysis confirming the contribution of collection sites and mosquito tissues to the mosquito fungal community assembly. (A) Fungal ASVs presented at the levels of phylum and order in mosquito tissues and breeding waters from each larval breeding site. ASV bar graphs were generated using the same methodology as the one described in the legend of Fig. 2 for the OTU analysis. Six fungal phyla were identified across all samples. ASVs assigned to the phylum Ascomycota accounted for the majority of the sequence reads (61%), followed by those belonging to the phyla Basidiomycota (29%), Mucoromycota (0.17%), Chytridiomycota (0.050%), Glomeromycota (0.004%), and Rozellomycota (0.004%). The remaining 9.71% of reads could not be classified past the kingdom Fungi but were included in all downstream analyses. Within the phylum Ascomycota, the fungal orders Eurotiales, Pleosporales, and Hypocreales were dominant and accounted for 22%, 20%, and 14% of the total reads, respectively. Within the phylum Basidiomycota, the order Malasseziales accounted for the majority of the reads (33%), followed by the orders Agaricales (19%) and Ustilaginales (11%). The majority of reads of the remaining four phyla belonged to the orders Mucorales (47%), Endogonales (21%), and Mortierellales (7.5%) within the phylum Mucoromycota and the orders Spizellomycetales (14%) and Rhizophlyctidales (3.5%) within the phylum Chytridiomycota. (B) Principal-coordinate analysis based on pairwise Bray-Curtis distances derived from the ASV data set. PCoA data visualization and statistical analyses based on fungal ASVs were performed as described in the legend of Fig. 3 for OTUs. The keys at the top left of each plot designate collection site by color, while the keys at the bottom left of each plot designate sample type (water versus mosquito gut or carcass) by symbol shape. Samples cluster significantly by larval breeding site (*F* = 5.1018; *P* < 0.001 [by PERMANOVA]). The results paralleled those obtained using the OTU-based approach and revealed significant differences in the mycobiota between samples from different sites. In addition, MANOVA and univariate ANOVAs further indicated significant differences along all three PCoA axes for site and the interaction of site and mosquito tissues, but samples were separated only by tissue along the first axis (see Table S6 in the supplemental material). (D) Alpha diversity indices of ASVs identified in the larval breeding water and mosquito tissue types across the larval breeding sites. (Left and middle) Estimated ASV richness (Chao1 index) (left) and observed ASV richness (Sobs index) (middle), between water and mosquito guts and carcasses across breeding sites. (Right) Shannon’s diversity index (*H*) between mosquito guts and carcasses across breeding sites. A Wilcoxon signed-rank test was used to determine statistical significance between samples. Statistical significance levels are indicated by **** (*P* < 0.0001). (E) Bray-Curtis distances using fungal ASVs between water and either the guts or carcasses of mosquito larvae across all breeding sites. Data visualization and statistical analysis were performed as described in the legend of Fig. 4 for OTUs. Connected lines depict matching mosquito gut and carcass samples for a given larval breeding site. Statistical significance was determined using separate Wilcoxon signed-rank tests for each site. The results parallel those observed using fungal OTUs where the fungal community associated with the mosquito carcasses was more dissimilar to that of the water than to that of the matched guts. Download FIG S3, PDF file, 0.3 MB. Copyright © 2021 Tawidian et al. 2021 Tawidian et al. https://creativecommons.org/licenses/by/4.0/ This content is distributed under the terms of the Creative Commons Attribution 4.0 International license.

### Mosquito feeding behavior and fungal mode of nutrition drive tissue-specific patterns of fungal community assembly.

**FIG 5:**
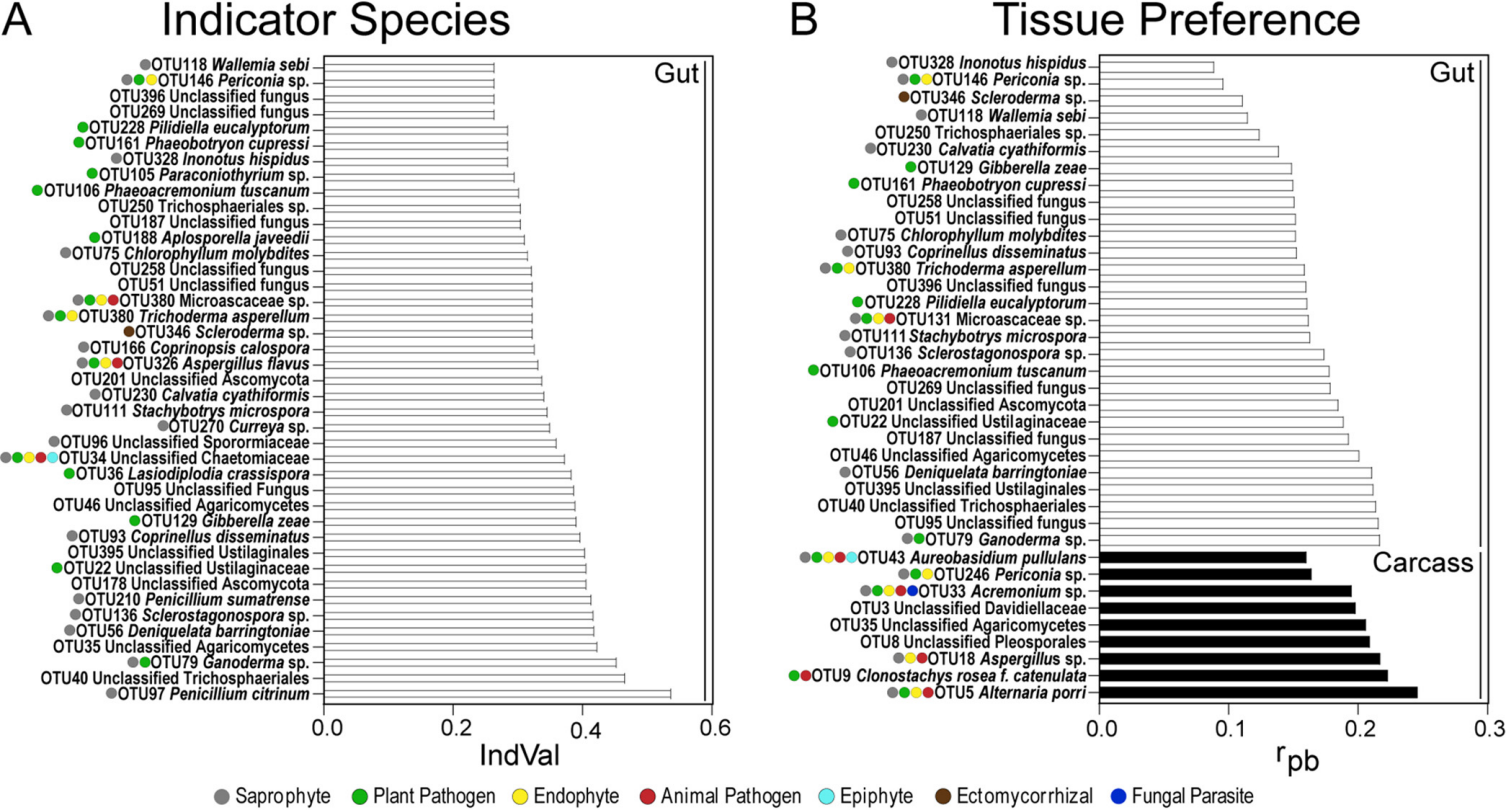
Indicator species and tissue preference of fungal OTUs between mosquito guts and carcasses across all larval breeding sites. (A) Characteristic fungal OTUs of mosquito guts across all breeding sites were identified using the indicator value index (IndVal) at a *P* value of 0.05 and with 9,999 permutations. (B) OTU tissue preferences between mosquito guts and carcasses across breeding sites were identified using the point-biserial correlation index (*r_pb_*) at a *P* value of 0.05 and with 9,999 permutations. Ecological guilds inferred by FUNGuild are organized from the most abundant to the least abundant guild.

To identify specific fungal OTUs that are differentially associated with the mosquito gut and carcass, we performed indicator taxon analyses using two different indices on the 149 OTUs that made up 80% of the total reads in the individual mosquito gut and carcass samples across all sites. Using the indicator value index (IndVal), we identified 41 indicator OTUs for mosquito guts across all breeding sites (Fig. 5A; Table S2). In contrast to the mosquito guts, the IndVal analysis identified no indicator OTUs of mosquito carcasses. The 41 mosquito gut indicator OTUs were assigned to six ecological guilds: saprophyte (34%), plant pathogen (25%), endophyte (8.9%), animal pathogen (5.3%), epiphyte (1.7%), and ectomycorrhiza (1.7%). The remaining 23% could not be assigned to ecological guilds.

10.1128/mSphere.00646-21.6 TABLE S2 Indicator value index (IndVal) calculations of 41 mosquito gut indicator species across larval breeding sites. Download Table S2, PDF file, 0.1 MB. Copyright © 2021 Tawidian et al. 2021 Tawidian et al. https://creativecommons.org/licenses/by/4.0/ This content is distributed under the terms of the Creative Commons Attribution 4.0 International license.

We then used the point-biserial correlation index (*r_pb_*) to determine the impact of fungal niche preference on differential fungal community assembly between mosquito guts and carcasses (Fig. 5B; Table S3). We found that 29 OTUs, all of which were also gut indicator OTUs, were significantly associated with mosquito guts (Table S3). The OTUs with mosquito gut preference were primarily assigned to the ecological guilds of saprophyte (32.4%), plant pathogen (21.6%), and endophyte (8.1%), followed by animal pathogen (2.7%) and ectomycorrhiza (2.7%). The remaining 32.4% of OTUs could not be assigned to any ecological guild. The *r_pb_* analysis identified nine OTUs that were significantly associated with mosquito carcasses (Table S3). The majority of these OTUs belonged to four ecological guilds: saprophyte (21.7%), endophyte (21.7%), animal pathogen (17.4%), and plant pathogen (17.4%), followed by fungal parasite (4.3%) and epiphyte (4.3%). The remaining 13% of OTUs could not be assigned to any ecological guild.

10.1128/mSphere.00646-21.7 TABLE S3 Point-biserial correlation (*r_pb_*) calculations of 32 OTUs with significant gut and carcass tissue preferences across larval breeding sites. Download Table S3, PDF file, 0.1 MB. Copyright © 2021 Tawidian et al. 2021 Tawidian et al. https://creativecommons.org/licenses/by/4.0/ This content is distributed under the terms of the Creative Commons Attribution 4.0 International license.

In addition to these indicator analyses, we also mined the data set for taxa with known entomopathogenic potential. We identified one OTU (OTU68) assigned to the genus *Beauveria*, which contains several known entomopathogens, and another (OTU9) assigned to Clonostachys rosea f. *catenulata*, which was significantly associated with mosquito carcasses in the *r_pb_* analysis. Sequences assigned to a member of the genus *Beauveria* were present in mosquito samples from 50% of sampled breeding sites. Within these sites, *Beauveria* sp. was found only sporadically (in 15 guts and 7 carcasses from 48 mosquito larvae in total), and only five larvae had OTU reads assigned to *Beauveria* in both guts and carcasses. The distribution of reads across these two tissue types in those five larvae did not show a clear trend, ranging from 10-fold-higher numbers of reads in guts than in carcasses and vice versa. Sequences assigned to *Clonostachys rosea* f. *catenulata* were present in only three of the nine sampled breeding sites. Within these sites, C. rosea f. *catenulata* was found in 24 gut and 23 carcass samples. In the 21 larvae where *C. rosea* f. *catenulata* was found in both guts and carcasses, the number of reads was on average 19-fold higher in the carcass than in the gut samples.

## DISCUSSION

This study reports the composition and diversity of fungal communities associated with *A. albopictus* larvae and their natural habitats on a small geographic scale. The larval habitat water and mosquito guts and carcasses sampled in our study harbored a diverse, uneven, and rich fungal community. Similar patterns of fungal diversity were reported in natural and artificial mosquito breeding sites sampled from different regions in Taiwan and in mosquito adults collected intercontinentally (28, 30). In our study, the fungal communities identified in breeding water and mosquito guts and carcasses were dominated by fungi of the phyla Ascomycota and Basidiomycota. This parallels previously reported fungal communities in water and organic substrates collected from tree holes and man-made containers (28, 42). Our results are not surprising and can be explained by the ubiquity of Ascomycota and Basidiomycota in freshwater ecosystems, including larval habitats, compared to the remaining fungal phyla (28, 43–46). The ITS2 primers (fITS7 and ITS4) that we used for amplification are commonly used in fungal community surveys (47–51) and readily detect members of the fungal phyla Ascomycota, Basidiomycota, Chytridiomycota, and Mucoromycota, as demonstrated by our mock fungal community. However, ITS2 primers show low species identification and discrimination in early-diverging fungal lineages (52, 53). Additionally, a considerable percentage (9%) of the OTUs assigned to unclassified fungi had to be manually reclassified upon closer inspection, most commonly to algae, which is consistent with previous studies reporting the amplification of algae by the primer pair fITS7 and ITS4 (54). Future studies using these primers should therefore also carefully inspect OTUs assigned to nonclassified fungi.

All previous studies that assessed the fungal community composition of mosquito larvae have used culture-dependent methods to isolate filamentous fungi and yeasts from whole larvae of several mosquito species (2, 18, 55, 56). A significant proportion of these isolated culturable fungi and yeasts were also identified in our OTU data set (17% at the species level and 44% at the genus level). The majority of these belong to the filamentous ascomycete genera Aspergillus, *Beauveria*, *Cladosporium*, *Penicillium*, and *Trichoderma* or the ascomycete yeast genus *Candida* and the basidiomycetous yeast genera Cryptococcus, *Rhodosporidium*, *Rhodotorula*, and *Trichosporon*. Similarly, 83% of the ascomycetous fungi thus far isolated from other aquatic insects, including caddisfly larvae, European nonbiting midge larvae, blackfly larvae, and adult aquatic beetles, are also present in our data set (57–59). As mentioned above, the overlap of fungal community composition within larval stages of various mosquito species and across other aquatic insects may be explained by the high prevalence of these taxa in freshwater habitats (28, 43–46).

We did not detect OTUs assigned to the genus *Smittium*, an early-diverging fungal lineage in the subphylum Kickxellomycotina, which contains several species of gut symbionts and pathogens of mosquito larvae (60–64). Despite its broad host range, to our knowledge, the genus *Smittium* has not been reported in *A. albopictus* larvae. It is thus possible that members of this genus are unable to colonize the gut of *A. albopictus* larvae. It is also possible that *Smittium* spp. do not persist in mosquito breeding grounds in Kansas and therefore remained undetected in our data set. However, an equally parsimonious explanation is a lack of detection due to the choice of primers that poorly capture early-diverging and basal fungal phyla (65). In future studies, we will therefore employ taxon-specific primers and microscopy techniques to assess the presence of *Smittium* spp. in *A. albopictus* larvae as described previously (66–68).

Our overall goal was to determine the drivers of fungal diversity associated with field-collected *A. albopictus* larvae. Our results show that the fungal communities in the larvae of this mosquito species largely reflect those of their breeding environments. To our knowledge, this is the first fungal data set with sampling at a local scale and sequences obtained from individual larvae from which the guts were dissected. Our results corroborate data identifying factors that shape the bacterial diversity in larvae of various mosquito species. Gimonneau et al. (6) showed that the bacterial communities of field-collected Anopheles gambiae and Anopheles coluzzii mosquito larvae largely overlapped those of the aquatic habitat, suggesting that the larval habitat is the major source of the microbiota assembly in mosquito larvae. Similar findings were also reported for the larval microbiota of several *Aedes*, *Anopheles*, and *Culex* species, including Aedes japonicus, Aedes triseriatus, Aedes aegypti, *A. albopictus*, Anopheles darlingi, Anopheles nuneztovari, Culex quinquefasciatus, and Culex restuans, collected from several man-made container breeding sites (7–9). We recognize that our study assessed the fungal community composition in only a single mosquito species. Hence, we cannot exclude the host as a significant factor shaping the fungal community in *A. albopictus* mosquito larvae. However, our results provide little evidence for filtering or enrichment of specific taxa that are shared across larval habitats, further supporting the dominant role of the larval aquatic habitat in the assembly of fungal communities associated with mosquito larvae.

Our results further show that the fungal communities in the mosquito larval gut and carcass differ. Although not identical to the larval breeding water, the gut fungal community was more similar to the environment than it was to the fungal community in the carcass. The similarity of the gut fungal community to the environment can be explained by the filter-feeding habit of mosquito larvae in general, which presumably captures a large portion of fungal taxa from the breeding water (41). *A. albopictus* larvae also display other feeding habits, including grazing and shredding on decaying leaf matter (69–71), which further contributes to the gut fungal diversity. This is supported by the results of the indicator species analysis where the gut indicator OTUs predominantly are saprophytes, endophytes, and pathogens that typically occur in plant tissues. To exemplify, one of the indicator OTUs belongs to the family Ustilaginaceae, which is composed of more than 1,200 obligately biotrophic fungal species that can infect more than 4,000 plant species (72). Members of this family can infect forage grasses and crops such as corn, barley, and wheat (72, 73). Several other plant-associated fungi that were abundant and frequent in the gut samples include Gibberella zeae, Phaeoacremonium tuscanum, and Phaeobotryon cupressi, taxa that likely represent putative pathogens of diverse plant crops, including wheat and grapevine, and trees (74–78). This further supports that the fungal diversity observed in the larval gut of mosquitoes is a consequence of feeding on plant material.

In contrast, the fungal diversity observed in mosquito carcasses, from which we had removed the heads to exclude any fungi captured on the mouth brushes during feeding, was lower and contained communities distinct from those in the larval guts and breeding water. Approximately one-half of the OTUs with a preference for carcass compared to gut were putative animal pathogens, suggesting that these OTUs either interact with the carcass by attaching to the cuticle or infect the carcass through the gut after ingestion (79–85). These included OTUs assigned to the species *C. rosea*, a fungal entomopathogen of several insect hosts, including leafhoppers, whiteflies, and alfalfa weevils (86–88), and Alternaria porri, which causes mortality in green apple aphids and delays hatch rates in egg masses of the European corn borer (89, 90). These two OTUs were also detected in gut samples, suggesting that ingestion might have led to systemic infection by these fungi. Overall, our data strongly suggest that in addition to mosquito feeding behavior, fungal ecology and niche preference further separate fungal communities in *A. albopictus* larval tissues.

Overall, our analyses found little additional evidence of *A. albopictus* infections with known fungal entomopathogens of mosquitoes. Of the 67 fungal species considered entomopathogenic and/or entomotoxigenic to mosquitoes (2), *Beauveria* sp. was the only previously described mosquito entomopathogen that we detected. This observation is consistent with a previous study describing the fungal community of field-collected *A. albopictus* adult females (30). However, the distribution of sequences in our data set provides little support for active mosquito infection by *Beauveria* in our collection sites. The sequences assigned to *Beauveria* sp. were rare and sporadic across the mosquito guts and even less commonly found in the carcass samples. Nevertheless, we were able to isolate a local strain of Beauveria bassiana from a single *A. albopictus* larva collected at site 5. Future studies with this isolate will test the efficacy of this strain to infect *A. albopictus* larvae.

In conclusion, this study provides fundamental insights into the broad range of encounters between mosquito larvae and fungi in the larval breeding water. Our results show that mosquito breeding water harbors a highly rich and diverse fungal community on a fine geographic scale, which drives the assembly of fungal communities that are associated with mosquito larvae. We further show the contribution of mosquito feeding behavior and fungal ecology to tissue-specific patterns of fungal community assembly. Future studies will have to assess whether these observed patterns can be generalized across different mosquito species and whether ontogeny further contributes to the assembly of fungal communities associated with mosquitoes.

## MATERIALS AND METHODS

### Sample collections.

*A. albopictus* L4 larvae and the corresponding water were sampled from a total of 10 breeding sites in Manhattan, KS, during 2017 and 2018: 2 naturally occurring mosquito breeding sites and 8 artificial (man-made) breeding sites consisting of plastic mosquito oviposition cups lined with heavyweight seed germination paper (Anchor Paper Co., MN, USA) (Fig. 1; see also Table S1 in the supplemental material). For the 2017 larval collections, mosquito larvae were kept in their respective environments, transported to the laboratory in labeled plastic containers (Bare Eco-Forward Rpet deli containers), and incubated at 27°C with 75% relative humidity (RH) for 24 h before larval gut dissections. For the 2018 larval collections, mosquito larvae were transported to the laboratory as described above for the 2017 collections but were dissected immediately upon arrival. Water samples were immediately stored at −80°C until nucleic acid extraction and sequencing.

### Mosquito dissections.

Mosquito larvae were surface washed six times with sterile MilliQ water to exclude any carryover from the breeding water not associated with the mosquito larvae. Larvae were then decapitated to exclude any transiently attached fungi from the environment on the mouth brushes and then dissected. Dissection of the larval gut from the body, referred to here as the mosquito carcass, was accomplished using flame-sterilized forceps and dissecting pins. Dissected gut and carcass samples were immediately frozen with liquid nitrogen and stored at −80°C until further processing. Finely cut, bleached nets were used as negative controls to screen for contamination during the dissection process. These negative controls were processed simultaneously with mosquito larva dissections.

### Sample preparation and Illumina MiSeq.

Total DNA was extracted using the DNeasy PowerSoil kit (MoBio Laboratory, Carlsbad, CA, USA) according to the manufacturer’s instructions, with minor modifications, from a total of 100 mosquito gut and carcass samples, 10 water samples filtered through 1-μm Nuclepore membranes (Whatman), and 7 dissection control samples. DNA samples were stored at −20°C until PCR amplification. Extracted DNA was quantitated using NanoDrop 2000/2000c spectrophotometers (Thermo Scientific, Waltham, MA, USA) and standardized to a concentration of 2 ng/μl. The fungal amplicon library was generated by triplicate PCR amplification using barcoded forward primer fITS7 (5′-GTGARTCATCGAATCTTTG-3′) and barcoded reverse primer ITS4 (5′-TCCTCCGCTTATTGATATGC-3′) according to a protocol described previously (91), with minor modifications. PCR with 20 ng of template DNA included an initial denaturation step for 30 s at 98°C and 35 cycles of denaturing, annealing, and extension at 98°C for 10 s, 54°C for 30 s, and 72°C for 1 min, followed by a final 72°C extension step for 9 min. The PCR negative control was certified nuclease-free sterile water. A mock fungal community was created with DNA from 10 fungal species belonging to different phyla to determine sequencing quality and the range of fungal taxon identifications (Table S4).

10.1128/mSphere.00646-21.8 TABLE S4 Taxonomic classification and reads of the assigned fungal operational taxonomic units (OTUs) in the larval breeding water and mosquito guts and carcasses. Download Table S4, TXT file, 1.6 MB. Copyright © 2021 Tawidian et al. 2021 Tawidian et al. https://creativecommons.org/licenses/by/4.0/ This content is distributed under the terms of the Creative Commons Attribution 4.0 International license.

Successful PCR amplification was determined by visualizing 5 μl of the PCR products on 1% agarose gels. The remaining 45 μl from each triplicate PCR was pooled and purified using Mag-Bind RXNPure plus (Omega Bio-Tek, Norcross, GA, USA). The clean amplicons from each sample were pooled at equal concentrations. Illumina MiSeq adaptors were ligated to the amplicon library using a Kapa library quantification kit for Illumina platforms (Kapa Biosystems, Wilmington, MA, USA), and sequences were generated using a MiSeq instrument (2 by 300 cycles; Illumina, San Diego, CA, USA) at the Kansas State University Integrated Genomics Facility (Manhattan, KS, USA).

### Sequence processing.

Paired-end sequences were processed using mothur (v.1.38.1) (92). Sequences with ambiguous bases, mismatches to primers, and homopolymers longer than 10 bp were removed. A total of 229,195 chimeric sequences were identified using the VSEARCH algorithm (93) and removed from the data set. Fungal sequences were assigned to taxa using the naive Bayesian classifier against the UNITE-curated International Nucleotide Sequence Database reference database (94, 95). During data processing through mothur, 191,081 sequences were either unassigned or assigned to unclassified plantae and the protozoan phyla Cercozoa and Ciliophora and removed from the data set. Fungal sequences were pairwise aligned to generate a distance matrix, which was clustered into OTUs using the average neighbor algorithm (unweighted pair group method using average linkages [UPGMA]) at a 97% similarity threshold. Low-abundance fungal OTUs represented by fewer than 10 sequences were removed from the data set. Finally, the National Center for Biotechnology Information (NCBI) Basic Local Alignment Search Tool (BLAST) (https://blast.ncbi.nlm.nih.gov/Blast.cgi) was used to identify any OTUs assigned to unclassified fungi. BLAST revealed 335 nonfungal OTU assignments containing 1,111,222 sequence reads, which were subsequently removed from the data set prior to downstream analyses. These OTUs belonged to the kingdoms Animalia, Eubacteria, Plantae, and Protista. The majority of the OTUs (41.2%) were assigned as algae, followed by plants (20.5%), protozoa (13.4%), insects (13.1%), uncultured eukaryotes and fish (8.95%), and bacteria (2.68%). Fungal OTUs present in the negative PCR or dissection controls (157 OTUs accounting for 349,100 sequences) were also removed from the data set, resulting in a final filtered fungal OTU data set consisting of 3,415 OTUs and 4,259,127 sequences.

### OTU data analysis.

To account for unequal sequencing depth while retaining rare taxa, we performed all downstream analyses on a modified filtered fungal data set (described above) that was not rarefied but excluded all paired mosquito samples for which we obtained <1,500 sequences from either the gut and/or carcass (96). This resulted in the elimination of all mosquito gut and carcass samples from site 3, two gut and carcass pairs from site 6, and a single gut and carcass pair from sites 2, 4, and 7 (Table S5). Fungal diversity and community composition analyses were conducted using mothur (v.1.38.1) (92). Nonparametric Wilcoxon signed-rank tests were then used to compare alpha diversity indices (OTU richness, Chao1, and Shannon’s *H* diversity indices) in the water and mosquito gut and carcass samples.

10.1128/mSphere.00646-21.9 TABLE S5 Indicator value index (IndVal) calculations of 64 mosquito gut indicator ASVs across the larval breeding sites. Download Table S5, PDF file, 0.1 MB. Copyright © 2021 Tawidian et al. 2021 Tawidian et al. https://creativecommons.org/licenses/by/4.0/ This content is distributed under the terms of the Creative Commons Attribution 4.0 International license.

To compare the fungal community compositions in the paired samples, we computed pairwise Bray-Curtis dissimilarity matrices and visualized them by principal-coordinate analyses (PCoAs) using mothur (v.1.38.1) (92). The compositional differences were inferred via PERMANOVA and MANOVA to determine whether fungal communities clustered based on site or mosquito tissue type (gut versus carcass). Additional ANOVAs were performed on significant MANOVA factors to determine which axis or axes were responsible for the clustering observed on the PCoA plots. To determine whether the guts or the carcasses were more similar to the breeding water, we performed Wilcoxon matched-pairs signed-rank tests comparing the Bray-Curtis distances between the breeding water and larval gut versus those of the breeding water and larval carcasses for each larva from a given site. To determine which OTUs may underlie the inferred community differences, we identified indicator OTUs that were disproportionately abundant in either mosquito guts or carcasses using the indicator value (IndVal) method (97). In addition, we determined the degree of preference of OTUs for mosquito guts or carcasses using the point-biserial correlation coefficient (*r_pb_*) (98). IndVal and *r_pb_* analyses were performed using the Indicspecies package implemented in R (99). For both IndVal and *r_pb_* analyses, 9,999 iterations were used to determine whether the OTUs were significantly associated with mosquito guts or carcasses. The FUNGuild database (100) was used to determine the ecological role of the fungal OTUs present in the heat map. Fungal OTUs were assigned to six ecological roles: animal pathogenic, ectomycorrhizal, endophytic, epiphytic, fungal parasitic, plant pathogenic, and saprophytic. Although the saprophytic OTUs were divided into dung saprophytic, litter saprophytic, plant saprophytic, soil saprophytic, and wood saprophytic, we assigned all saprophytic OTUs as saprophytes in our analyses. Wilcoxon signed-rank tests were performed using GraphPad Prism version 8.4.3 for Windows (GraphPad Software, San Diego, CA, USA), whereas MANOVAs and ANOVAs were performed using R (http://www.r-project.org/).

### ASV data analysis and comparison with OTU data analysis results.

Paired-end sequences were processed using mothur (v.1.44.3) (92). After pairwise alignment, sequences were preclustered into amplicon sequence variants (ASVs) at a threshold of a 2-nucleotide difference. A total of 229,195 chimeric sequences were identified using the VSEARCH algorithm (93) and removed from the data set. The resulting data set contained 117,162 ASVs and 7,833,850 sequence reads. The additional nonfungal ASVs (30,925 ASVs accounting for 3,498,860 sequences) associated with the 335 nonfungal OTUs identified previously with BLAST were removed from the data set using mothur (v.1.44.3). These ASVs belonged to the kingdoms Animalia, Eubacteria, Plantae, and Protista. The majority of nonfungal ASVs (48.8%) were assigned as algae, followed by bacteria (23.4%), protozoa (10.07%), insects (5.76%), uncultured eukaryotes and fish (4.89%), and plants (4.60%). Fungal ASVs present in the negative PCR or dissection controls (4,048 ASVs accounting for 407,902 sequences) were also removed from the data set. In addition, low-abundance fungal ASVs represented by fewer than 10 sequences were removed from the data set, resulting in a final filtered fungal data set consisting of 7,798 ASVs and 4,180,758 sequences. Similar to the OTU data set, rarefaction curves saturated or nearly saturated at 5,000 sequences for most samples, indicating that the fungal diversity was captured in our sampling regardless of the methodology used to infer community composition (Text S1). This is consistent with the high Good’s coverage estimates that we observed across all samples (0.999 ± 0.001 standard deviation).

10.1128/mSphere.00646-21.1 TEXT S1 Amplicon sequence variant analysis methodology and results. Download Text S1, PDF file, 0.1 MB. Copyright © 2021 Tawidian et al. 2021 Tawidian et al. https://creativecommons.org/licenses/by/4.0/ This content is distributed under the terms of the Creative Commons Attribution 4.0 International license.

Consistent with some of the previous reports that had compared ASV and OTU analyses of bacterial 16S or fungal ITS data sets (101, 102), the overall ecological patterns that we observed did not differ between these two methods. We chose to present the results of the OTU data set in the text, as all previous ITS data sets extracted from mosquito samples have used OTUs to define mosquito-associated fungal communities. The parallel analyses using ASVs as well as a detailed comparison of the results obtained with both data sets can be found in the supplemental material.

### Data availability.

Paired sequence data (.fastq files) are available in the NCBI Sequence Read Archive under BioProject accession number PRJNA634912.

10.1128/mSphere.00646-21.10 TABLE S6 Point-biserial correlation (*r_pb_*) calculations of 48 ASVs with significant gut preference and 7 ASVs with significant carcass preference across larval breeding sites. Download Table S6, PDF file, 0.1 MB. Copyright © 2021 Tawidian et al. 2021 Tawidian et al. https://creativecommons.org/licenses/by/4.0/ This content is distributed under the terms of the Creative Commons Attribution 4.0 International license.
