# Supplementary figures and images for: Host-Environment Interplay Shapes Fungal Diversity in Mosquitoes
Source: mSphere. 2021 Sep 29;6(5):e00646-21. doi: 10.1128/mSphere.00646-21 (PMC8550294; doi:10.1128/mSphere.00646-21)

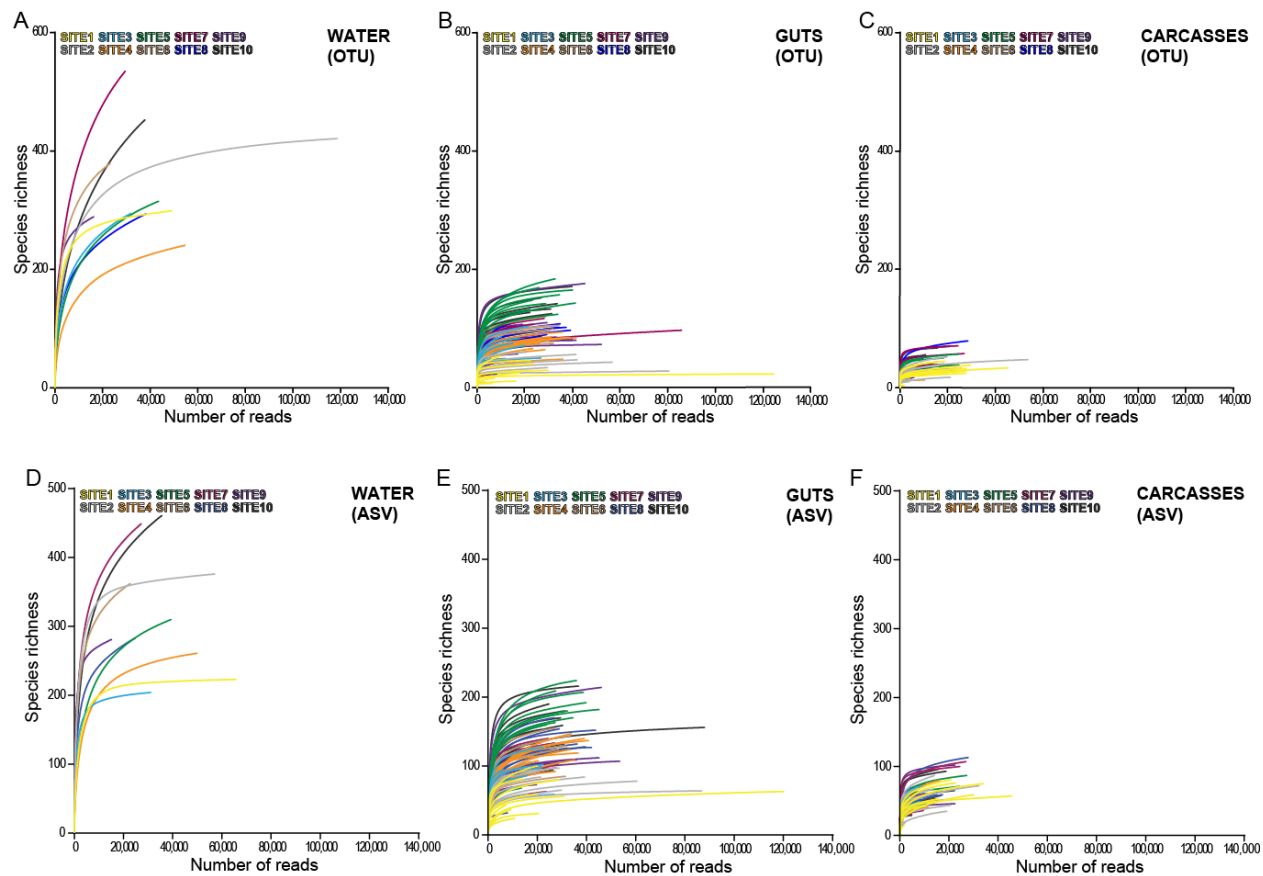

Supplement: FIG S1 [file msphere.00646-21-sf001.pdf]

A

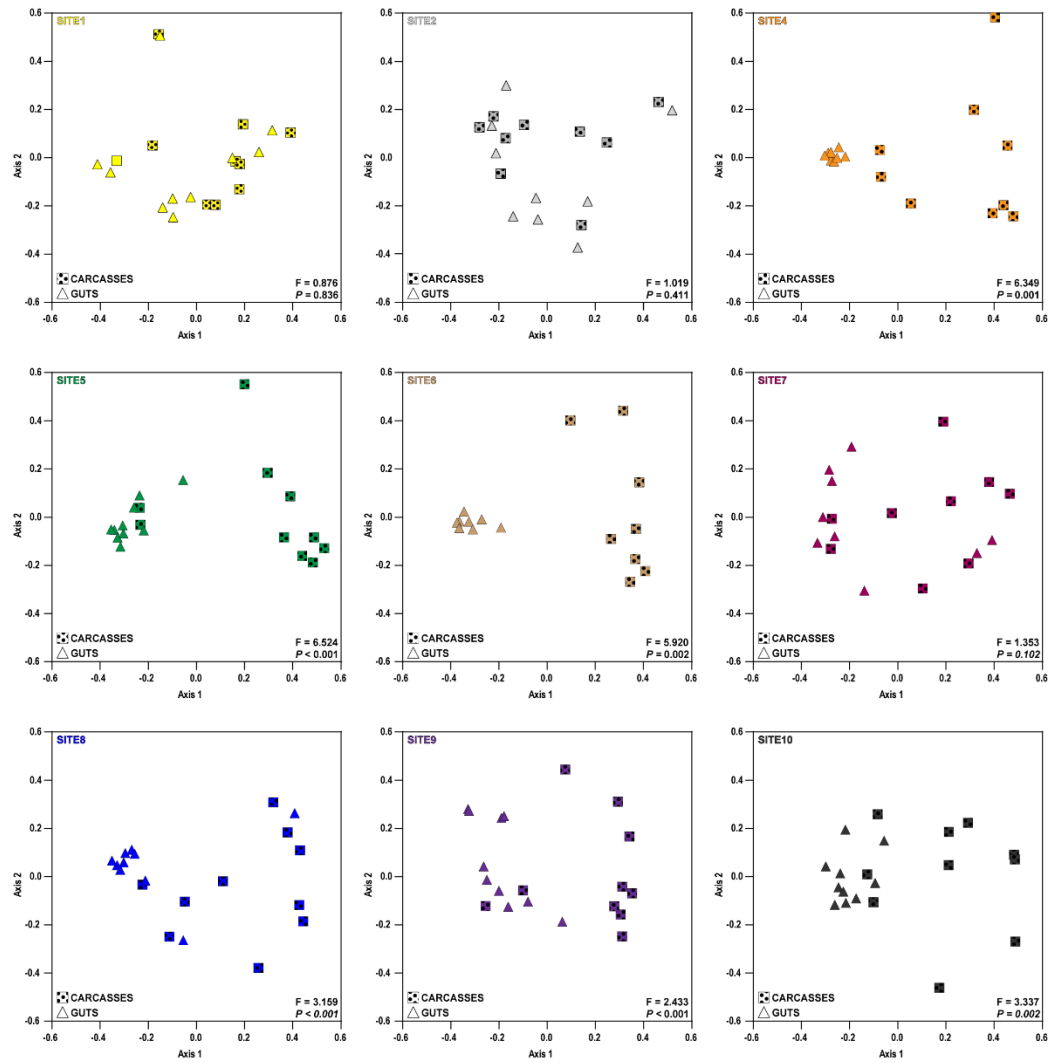

B

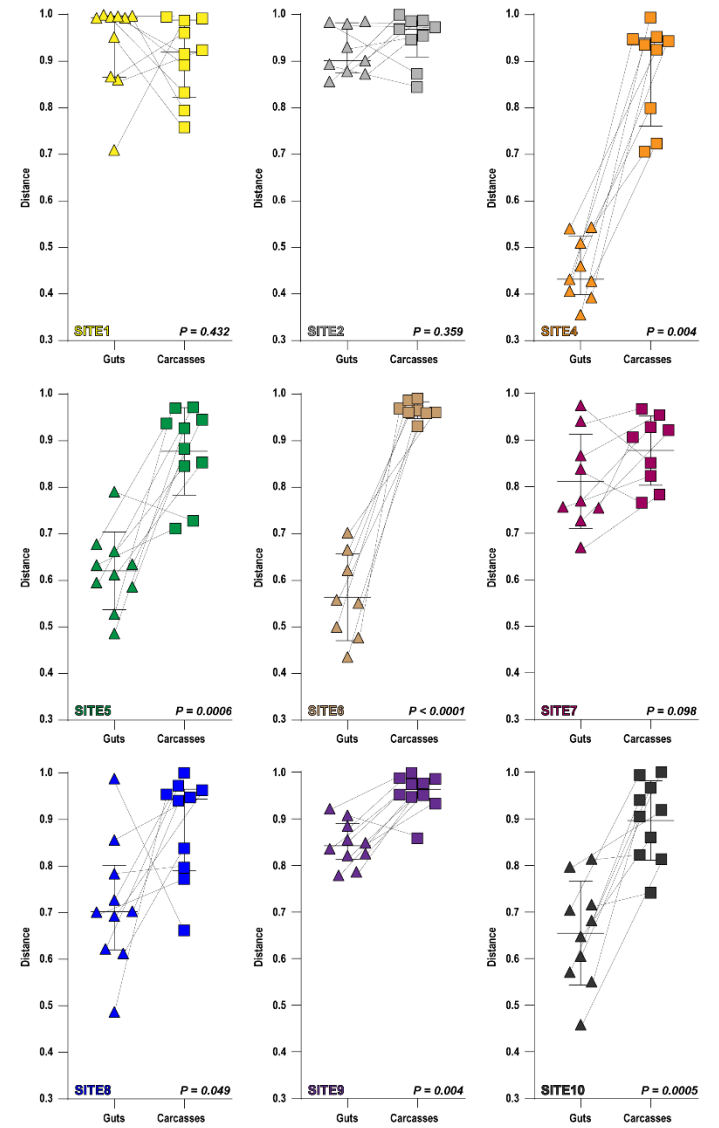

Supplement: FIG S2 [file msphere.00646-21-sf002.pdf]

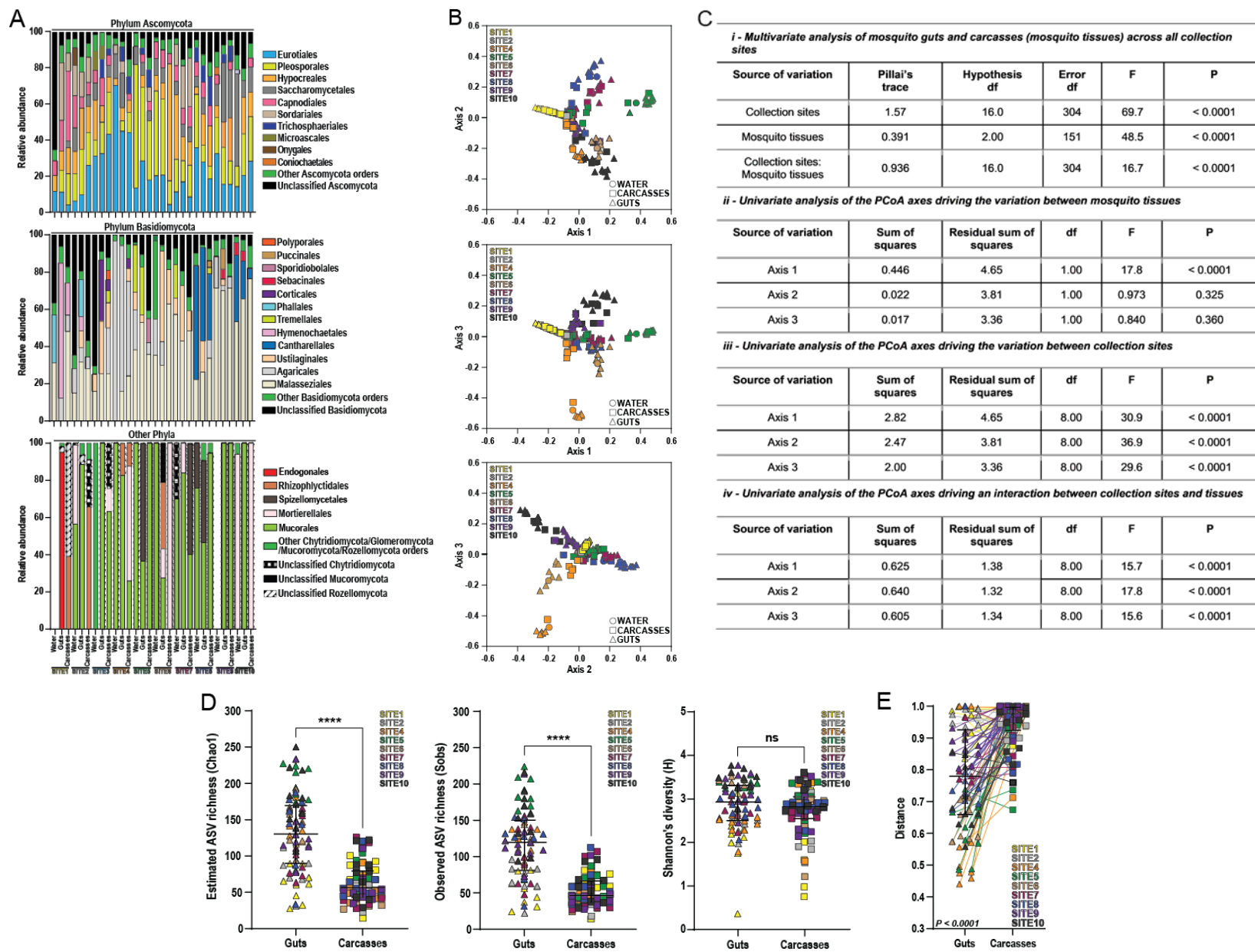

Supplement: FIG S3 [file msphere.00646-21-sf003.pdf]
