# Supplementary material for: Host-Environment Interplay Shapes Fungal Diversity in Mosquitoes
Source: mSphere. 2021 Sep 29;6(5):e00646-21. doi: 10.1128/mSphere.00646-21 (PMC8550294; doi:10.1128/mSphere.00646-21)
Supplement: TABLE S1 [file msphere.00646-21-st001.pdf]

Table S1. Alpha diversity indices of the larval breeding water and mosquito guts and carcass across the larval breeding sites.

Larval breeding site	Collection date	Lat, Long	Type of larval breeding site	Mosquito larvae	Sample type	Total # of sequences	Observed OTU richness (Sobs)	Estimated OTU richness (Chao1)	Shannon's diversity (H)
1	09/19/2017	N 39°10'53.0", W 96°35'35.5"	Natural breeding site		Water	49,086	299	337	3.67
				1	Gut	16,650	11.0	11.5	1.84
					Carcass	1,710	4.00	4.00	0.692
				2	Gut	7,354	16.0	17.0	1.94
					Carcass	18,885	48.0	52.2	2.40
				3	Gut	18,148	25.0	32.0	2.25
					Carcass	29,932	41.0	50.0	1.88
				4	Gut	6,873	20.0	20.5	2.14
					Carcass	27,899	30.0	37.0	1.99
				5	Gut	9,816	53.0	62.4	2.70
					Carcass	5,121	21.0	22.5	2.04
				6	Gut	6,355	7.00	7.00	1.41
					Carcass	27,755	26.0	28.0	2.46
				7	Gut	22,732	46.0	46.2	2.32
					Carcass	45,709	36.0	58.5	0.686
				8	Gut	126,191	23.0	23.5	0.462
					Carcass	28,054	35.0	37.5	2.31
				9	Gut	24,002	40.0	47.5	2.65
					Carcass	7,006	41.0	50.3	2.81
				10	Gut	30,147	29.0	29.5	2.22
					Carcass	17,014	30.0	40.5	2.22
2	09/25/2017	N 39°10'56.1", W 96°34'49.1"	Natural breeding site		Water	118,604	421	435	3.97
				1	Gut	10,788	38.0	39.5	1.72
					Carcass	3,939	11.0	11.0	1.60
				2	Gut	57,441	43.0	48.0	2.39
					Carcass	2,356	14.0	14.0	1.79
				3	Gut	14,084	29.0	32.0	1.98
					Carcass	16,407	40.0	43.8	2.69
				4	Gut	3,504	9.0	9.0	1.44
					Carcass	21,326	19.0	21.0	1.53
				5	Gut	30,093	34.0	62.0	2.43
					Carcass	19,899	56.0	67.0	2.11
				6	Gut	10,400	27.0	30.0	2.31
					Carcass	6,236	38.0	38.8	3.08
				7	Gut	42,482	47.0	56.3	2.37
					Carcass	54,147	51.0	56.6	1.65
				8	Gut	42,142	56.0	65.3	2.59
					Carcass	11,381	30.0	31.5	2.46
				9	Gut	21,147	60.0	67.5	2.86
					Carcass	25,981	35.0	36.5	2.97
4	08/13/2018	N 39°12'01.6", W 96°33'20.1"	Oviposition cup		Water	54,935	241	288	2.26
				1	Gut	36,544	94.0	99.0	2.16
					Carcass	10,684	14.0	14.0	1.14
				2	Gut	23,885	66.0	79.2	2.15
					Carcass	11,536	32.0	35.0	2.27
				3	Gut	35,845	82.0	90.7	2.21
					Carcass	3,676	25.0	30.0	2.52
				4	Gut	42,294	84.0	95.2	2.32
					Carcass	4,763	27.0	34.0	2.47

				5	Gut	22,950	75.0	113	2.21
					Carcass	16,084	47.0	56.3	2.51
				6	Gut	36,705	48.0	49.2	1.69
					Carcass	3,584	20.0	21.0	2.26
				7	Gut	27,240	77.0	101	2.15
					Carcass	4,935	33.0	34.5	2.61
				8	Gut	31,945	86.0	97.1	2.35
					Carcass	4,079	19.0	20.0	2.35
				9	Gut	28,968	64.0	91.5	2.15
					Carcass	7,503	40.0	47.5	3.01
5	09/12/2018	N 39°11'35.5", W 96°34'15.7"	Oviposition cup		Water	43,501	315	368	2.34
				1	Gut	41,773	143	185	3.09
					Carcass	4,534	19.0	19.0	2.16
				2	Gut	33,268	184	217	3.38
					Carcass	3,087	20.0	30.0	2.05
				3	Gut	29,338	142	148	3.18
					Carcass	8,514	53.0	66.8	2.87
				4	Gut	22,566	132	140	2.97
					Carcass	4,725	29.0	30.0	2.76
				5	Gut	35,017	157	184	3.38
					Carcass	8,993	37.0	39.5	2.34
				6	Gut	23,661	148	164	3.32
					Carcass	24,930	41.0	46.0	0.920
				7	Gut	12,330	49.0	50.5	3.11
					Carcass	24,794	61.0	64.5	2.57
				8	Gut	26,461	169	188	2.99
					Carcass	3,912	25.0	28.0	2.70
				9	Gut	34,380	124	139	3.02
					Carcass	18,429	54.0	69.0	2.83
				10	Gut	40,792	165	170	3.03
					Carcass	4,500	27.0	34.5	2.28
6	09/12/2018	N 39°10'56.2", W 96°34'48.7"	Oviposition cup		Water	23,222	379	427	3.47
				1	Gut	31,398	105	118	3.12
					Carcass	3,689	26.0	26.3	2.32
				2	Gut	18,279	72.0	75.0	2.89
					Carcass	3,147	22.0	28.0	2.34
				3	Gut	29,311	120	165	3.19
					Carcass	1,842	17.0	17.0	2.45
				4	Gut	34,573	105	112	2.96
					Carcass	4,183	17.0	17.0	2.07
				5	Gut	18,643	78.0	104	2.89
					Carcass	2,291	18.0	24.0	2.22
				6	Gut	32,571	75.0	93.0	2.80
					Carcass	2,062	22.0	22.0	2.68
				7	Gut	32,542	99.0	132	3.10
					Carcass	20,354	28.0	34.0	1.18
				8	Gut	33,480	105	114	3.24
					Carcass	5,829	35.0	35.0	3.05
7	09/14/2018	N 39°11'45.1", W 96°35'23.1"	Oviposition cup		Water	29,567	535	652	3.90
				1	Gut	86,886	97.0	124	2.77
					Carcass	3,893	26.0	27.5	2.52
				2	Gut	5,549	23.0	24.5	2.21
					Carcass	2,281	11.0	11.0	1.93
				3	Gut	16,184	45.0	45.6	2.41
					Carcass	27,136	62.0	83.0	2.64
				4	Gut	17,879	75.0	90.0	2.88
					Carcass	16,020	42.0	47.0	2.12
				5	Gut	26,510	90.0	101	2.29
					Carcass	24,512	76.0	76.8	3.34
				6	Gut	8,317	88.0	96.3	2.98

					Carcass	1,589	17.0	17.0	2.10
				7	Gut	16,520	105	120	3.09
					Carcass	4,575	26.0	29.0	2.68
				8	Gut	4,863	23.0	23.5	2.31
					Carcass	23,646	30.0	31.5	1.76
				9	Gut	23,872	107	113	2.49
					Carcass	5,573	26.0	28.0	2.34
8	09/21/2018	N 39°11'40.1", W 96°34'29.3"	Oviposition cup		Water	38,335	294	365	3.23
				1	Gut	28,287	121	151	2.71
					Carcass	6,118	32.0	37.0	2.50
				2	Gut	35,063	102	117	2.49
					Carcass	5,236	20.0	20.0	2.32
				3	Gut	40,767	83.0	96.2	2.69
					Carcass	3,331	20.0	20.3	2.31
				4	Gut	37,806	102	110	2.40
					Carcass	13,276	36.0	39.3	2.46
				5	Gut	11,306	46.0	61.0	2.51
					Carcass	18,248	30.0	33.0	2.11
				6	Gut	33,546	85.0	91.4	2.40
					Carcass	5,232	32.0	42.5	2.64
				7	Gut	8,403	22.0	22.0	1.89
					Carcass	28,747	85.0	103	2.75
				8	Gut	29,840	90.0	123	2.61
					Carcass	9,825	54.0	61.0	2.65
				9	Gut	39,794	97.0	107	2.73
					Carcass	7,732	38.0	41.0	2.88
				10	Gut	35,418	108	138	2.70
					Carcass	5,780	19.0	29.0	1.36
9	09/21/2018	N 39°10'56.2", W 96°34'48.7"	Oviposition cup		Water	16,275	289	331	4.39
				1	Gut	45,827	176	198	3.40
					Carcass	8,999	35.0	36.5	2.85
				2	Gut	14,724	93.0	98.0	3.69
					Carcass	10,096	36.0	37.5	2.59
				3	Gut	52,929	73.0	79.0	1.88
					Carcass	6,287	25.0	25.0	2.50
				4	Gut	34,127	85.0	95.5	3.44
					Carcass	17,050	29.0	35.0	1.90
				5	Gut	29,796	110	136	3.25
					Carcass	7,641	27.0	27.5	1.90
				6	Gut	27,437	86.0	96.0	2.91
					Carcass	4,635	20.0	20.5	2.24
				7	Gut	42,186	80.0	81.0	2.96
					Carcass	21,009	75.0	77.5	3.24
				8	Gut	19,433	107	113	3.20
					Carcass	22,289	42.0	42.5	2.24
				9	Gut	25,959	97.0	101	3.27
					Carcass	2,830	18.0	18.0	2.23
				10	Gut	17,285	57.0	60.0	3.10
					Carcass	2,896	39.0	46.5	3.20
10	09/21/2018	N 39°10'56.2", W 96°34'49.0"	Oviposition cup		Water	37,901	453	598	2.88
				1	Gut	27,520	152	179	3.52
					Carcass	8,138	58.0	58.8	3.43
				2	Gut	22,195	116	118	3.22
					Carcass	15,770	72.0	72.2	3.25
				3	Gut	22,426	127	138	3.16
					Carcass	2,896	22.0	23.5	2.57
				4	Gut	31,497	98.0	116	3.06
					Carcass	14,175	43.0	48.0	2.63
				5	Gut	25,278	72.0	105	2.76
					Carcass	19,604	73.0	79.0	3.30

				6	Gut	31,289	133	138	3.33
					Carcass	10,938	60.0	67.2	3.11
				7	Gut	34,234	142	159	3.42
					Carcass	21,803	42.0	43.5	2.53
				8	Gut	31,769	125	146	3.38
					Carcass	8,444	25.0	25.0	2.59
				9	Gut	26,216	76.0	98.5	3.43
					Carcass	8,120	25.0	25.5	2.49
				10	Gut	40,390	171	182	3.39
					Carcass	18,781	44.0	49.0	2.90
