# Supplementary material for: Host-Environment Interplay Shapes Fungal Diversity in Mosquitoes
Source: mSphere. 2021 Sep 29;6(5):e00646-21. doi: 10.1128/mSphere.00646-21 (PMC8550294; doi:10.1128/mSphere.00646-21)
Supplement: TABLE S2 [file msphere.00646-21-st002.pdf]

Table S2. Indicator value index (IndVal) calculations of 41 mosquito gut Indicator species across larval breeding sites.

OTUID	Taxa assignment	Ecological guild	IndVal	A_{ind}	B_{pa}
Otu00097	*Penicillium citrinum*	Saprophyte	0.539	0.85	0.341
Otu00040	Unclassified Trichosphaeriales	-	0.468	0.98	0.224
Otu00079	*Ganoderma* sp.	Saprophyte, Plant pathogen	0.455	0.88	0.235
Otu00035	Unclassified Agaricomycetes	-	0.425	0.639	0.282
Otu00056	*Deniquelata barringtoniae*	Saprophyte	0.42	0.882	0.2
Otu00136	Unclassified Sclerostagonospora	Saprophyte	0.419	0.785	0.224
Otu00210	*Penicillium sumatrense*	Saprophyte	0.416	0.866	0.2
Otu00022	Unclassified Ustilaginaceae	Plant pathogen	0.408	0.944	0.176
Otu00178	Unclassified Ascomycota	-	0.408	0.709	0.235
Otu00395	Unclassified Ustilaginales	-	0.406	1	0.165
Otu00093	*Coprinellus disseminatus*	Saprophyte	0.399	0.969	0.165
Otu00129	*Gibberella zeae*	Plant pathogen	0.393	0.939	0.165
Otu00046	Unclassified Agaricomycetes	-	0.391	1	0.153
Otu00095	Unclassified Fungus	-	0.389	0.86	0.176
Otu00036	*Lasiodiplodia crassispora*	Plant pathogen	0.385	0.741	0.2
Otu00034	Unclassified Chaetomiaceae	Saprophyte, Plant pathogen, Endophyte, Animal pathogen, Epiphyte	0.375	0.702	0.2
Otu00096	Unclassified Sporormiaceae	Saprophyte	0.362	0.855	0.153
Otu00270	*Curreya* sp.	Saprophyte	0.352	0.702	0.176
Otu00111	*Stachybotrys microspora*	Saprophyte	0.348	0.857	0.141
Otu00230	*Calvatia cyathiformis*	Saprophyte	0.343	1	0.118
Otu00201	Unclassified Ascomycota	-	0.34	0.981	0.118
Otu00326	*Aspergillus flavus*	Saprophyte, Plant pathogen, Endophyte, Animal pathogen	0.334	0.789	0.141
Otu00166	*Coprinopsis calospora*	Saprophyte	0.328	0.831	0.129
Otu00051	Unclassified Fungus	-	0.325	0.995	0.106
Otu00131	Unclassified Microascaceae	Saprophyte, Plant pathogen, Animal pathogen, Endophyte	0.325	0.898	0.118
Otu00346	*Scleroderma* sp.	Ectomycorrhizal	0.325	1	0.106
Otu00380	*Trichoderma asperellum*	Saprophyte, Plant pathogen, Endophyte	0.325	1	0.106
Out00258	Unclassified Fungus	-	0.324	0.894	0.118
Otu00075	*Chlorophyllum molybdites*	Saprophyte	0.318	0.78	0.129
Otu00188	*Aplosporella javeedii*	Plant pathogen	0.313	0.834	0.118
Otu00187	Unclassified Fungus	-	0.307	1	0.094
Otu00250	Unclassified Trichosphaeriales	-	0.307	1	0.094
Otu00106	*Phaeoacremonium tuscanum*	Plant pathogen	0.304	0.872	0.106
Otu00105	*Paraconiothyrium* sp	Plant pathogen	0.297	0.836	0.106
Otu00161	*Phaeobotryon cupressi*	Plant pathogen	0.287	1	0.082
Otu00228	*Pilidiella eucalyptorum*	Plant pathogen	0.287	1	0.082
Otu00328	*Inonotus hispidus*	Saprophyte	0.287	1	0.082
Otu00118	*Wallemia sebi*	Saprophyte	0.266	0.999	0.071
Otu00146	*Periconia* sp.	Saprophyte, Plant pathogen, Endophyte	0.266	1	0.071
Otu00269	Unclassified Fungus	-	0.266	1	0.071
Otu00396	Unclassified Fungus	-	0.266	1	0.071
