# Supplementary material for: Host-Environment Interplay Shapes Fungal Diversity in Mosquitoes
Source: mSphere. 2021 Sep 29;6(5):e00646-21. doi: 10.1128/mSphere.00646-21 (PMC8550294; doi:10.1128/mSphere.00646-21)
Supplement: TABLE S3 [file msphere.00646-21-st003.pdf]

Table S3. Point-biserial correlation (r_{pb}) calculations of 32 OTUs with significant gut and carcass tissue preferences across larval breeding sites.

OTUID	Taxa	Ecological guild	r_{pb}	p-value
**Gut**				
Otu00079	*Ganoderma* sp.	Plant pathogen, Saprophyte	0.217	0.0019
Otu00051	Unclassified Fungus	-	0.216	0.0049
Otu00040	Unclassified Trichosphaeriales	-	0.214	0.0002
Otu00395	Unclassified Ustilaginales	-	0.212	0.0001
Otu00056	*Deniquelata barringtoniae*	Saprophyte	0.211	0.0031
Otu00046	Unclassified Agaricomycetes	-	0.201	0.0004
Otu00095	Unclassified Fungus	-	0.193	0.0071
Otu00022	Unclassified Ustilaginaceae	-	0.189	0.004
Otu00201	Unclassified Ascomycota	-	0.185	0.0032
Out00258	Unclassified Fungus	-	0.179	0.0131
Otu00106	*Phaeoacremonium tuscanum*	Plant pathogen	0.178	0.0164
Otu00136	*Sclerostagonospora* sp.	Saprophyte	0.174	0.0203
Otu00111	*Stachybotrys microspora*	Saprophyte	0.163	0.0315
Otu00131	Unclassified Microascaceae	Animal pathogen, Endophyte, Plant pathogen, Saprophyte	0.162	0.0142
Otu00228	*Pilidiella eucalyptorum*	Plant pathogen	0.161	0.0134
Otu00269	Unclassified Fungus	-	0.16	0.0317
Otu00380	*Trichoderma asperellum*	Endophyte, Plant pathogen, Saprophyte	0.159	0.0029
Otu00093	*Coprinellus disseminatus*	Saprophyte	0.153	0.0024
Otu00075	*Chlorophyllum molybdites*	Saprophyte	0.152	0.048
Otu00187	Unclassified Fungus	-	0.152	0.0075
Otu00396	Unclassified Fungus	-	0.151	0.0317
Otu00161	*Phaeobotryon cupressi*	Plant pathogen	0.15	0.0127
Otu00129	*Gibberella zeae*	Plant pathogen	0.149	0.0272
Otu00230	*Calvatia cyathiformis*	Saprophyte	0.139	0.0018
Otu00250	Unclassified Trichosphaeriales	-	0.124	0.0048
Otu00118	*Wallemia sebi*	Saprophyte	0.115	0.0288
Otu00346	*Scleroderma* sp.	Ectomycorrhizal	0.111	0.0029
Otu00146	*Periconia* sp.	Endophyte, Plant pathogen, Saprophyte	0.096	0.0278
Otu00328	*Inonotus hispidus*	Saprophyte	0.089	0.0132
**Carcass**				
Otu00005	*Alternaria porri*	Saprophyte, Plant pathogen, Endophyte, Animal pathogen	0.246	0.0002
Otu00009	*Clonostachys rosea f. catenulata*	Plant pathogen	0.223	0.0002
Otu00018	*Aspergillus* sp.	Saprophyte, Endophyte, Animal pathogen	0.217	0.0027
Otu00008	Unclassified Pleosporales	-	0.209	0.0058
Otu00035	Unclassified Agaricomycetes	-	0.206	0.0013
Otu00003	Unclassified Davidiellaceae	-	0.198	0.005
Otu00033	*Acremonium* sp.	Saprophyte, Plant pathogen, Endophyte, Animal pathogen, Fungal parasite	0.195	0.0006
Otu00246	*Periconia* sp.	Saprophyte, Plant pathogen, Endophyte	0.164	0.003
Otu00043	*Aureobasidium pullulans*	Animal Pathogen-Endophyte-Epiphyte-Plant Pathogen-Undefined Saprotroph	0.16	0.0262
