# Supplementary material for: Host-Environment Interplay Shapes Fungal Diversity in Mosquitoes
Source: mSphere. 2021 Sep 29;6(5):e00646-21. doi: 10.1128/mSphere.00646-21 (PMC8550294; doi:10.1128/mSphere.00646-21)
Supplement: TABLE S5 [file msphere.00646-21-st005.pdf]

Table S5. Indicator value index (IndVal) calculations of 64 mosquito gut Indicator ASVs across the larval breeding sites.

ASVID	Taxa assignment	Ecological guild	IndVal	A_{ind}	B_{pa}	p-value
Asv90080	*Penicillium* sp.	Saprophyte	0.548	0.798	0.376	0.0002
Asv92426	Unclassified Fungus	-	0.514	0.865	0.306	0.0002
Asv90124	*Saccharomyces eubayanus*	Saprophyte	0.445	0.800	0.247	0.0002
Asv113293	*Aspergillus* sp.	Saprophyte	0.407	0.938	0.176	0.0007
Asv106764	*Cordyceps bassiana*	Plant pathogen, Symbiont	0.400	0.850	0.188	0.0068
Asv91545	*Zopfiella marina*	Saprophyte	0.391	1.00	0.153	0.0002
Asv90705	Unclassified Davidiellaceae	-	0.389	0.643	0.235	0.0316
Asv109290	*Malassezia* sp.	Saprophyte, Plant pathogen	0.384	0.738	0.200	0.0295
Asv99680	Unclassified Sebacinales	-	0.381	0.883	0.165	0.0008
Asv90082	*Saccharomyces eubayanus*	Saprophyte	0.377	0.864	0.165	0.0085
Asv121166	*Thanatephorus cucumeris*	Plant pathogen	0.377	0.864	0.165	0.003
Asv98754	*Aspergillus piperis*	Saprophyte	0.377	0.929	0.153	0.0018
Asv90683	Unclassified Malasseziales	-	0.376	0.667	0.212	0.0308
Asv090134	*Malassezia restricta*	Saprophyte, Plant pathogen	0.376	1.00	0.141	0.0004
Asv90938	*Paraconiothyrium* sp.	Saprophyte	0.376	1.00	0.141	0.0002
Asv118130	*Malassezia restricta*	Saprophyte, Plant pathogen	0.369	0.771	0.176	0.0371
Asv090182	*Saccharomyces* sp.	Saprophyte	0.368	0.676	0.200	0.0351
Asv100048	Unclassified Montagnulaceae	Saprophyte, Plant pathogen	0.360	0.919	0.141	0.009
Asv99655	Unclassified Montagnulaceae	Saprophyte, Plant pathogen	0.360	1.00	0.129	0.0011
Asv128755	*Malassezia restricta*	Saprophyte, Plant pathogen	0.360	1.00	0.129	0.0008
Asv94461	Unclassified Ustilaginaceae	Plant pathogen	0.354	0.968	0.129	0.0022
Asv106556	*Cordyceps bassiana*	Plant pathogen, Symbiont	0.344	0.773	0.153	0.0303
Asv97619	*Coprinellus xanthothrix*	Saprophyte	0.343	1.00	0.117	0.0023
Asv97302	Unclassified Montagnulaceae	Saprophyte, Plant pathogen	0.337	0.876	0.129	0.0111
Asv90207	Unclassified Ascomcyota	-	0.33	0.769	0.141	0.0365
Asv90198	*Malassezia globosa*	Saprophyte, Plant pathogen	0.325	1.00	0.106	0.003
Asv96534	*Cytospora* sp.	Saprophyte, Plant pathogen, Symbiont	0.325	1.00	0.106	0.0024
Asv121705	*Cordyceps bassiana*	Plant pathogen, Symbiont	0.325	1.00	0.106	0.0026
Asv97308	Unclassified Montagnulaceae	-	0.317	0.854	0.118	0.022
Asv106555	Unclassified Montagnulaceae	Saprophyte, Plant pathogen	0.322	0.881	0.118	0.0255
Asv90169	*Aspergillus brasiliensis*	Saprophyte	0.321	0.878	0.118	0.0079
Asv90086	*Saccharomyces eubayanus*	Saprophyte	0.317	0.856	0.118	0.0222
Asv97315	*Deniquelata barringtoniae*		0.317	0.854	0.118	0.022
Asv106925	Unclassified Eurotiomycetes	-	0.309	0.901	0.106	0.0169
Asv106391	Unclassified Montagnulaceae	Saprophyte, Plant pathogen	0.307	1.00	0.094	0.0072
Asv128734	*Malassezia restricta*	Saprophyte, Plant pathogen	0.307	1.00	0.094	0.0053
Asv90589	Unclassified Trichosphaeriales	-	0.307	1.00	0.094	0.007
Asv97357	*Gibberella intricans*	Plant pathogen	0.307	1.00	0.094	0.0062
Asv91643	*Trichoderma* sp.	Saprophyte	0.307	1.00	0.094	0.0069
Asv106380	Unclassified Montagnulaceae	Saprophyte, Plant pathogen	0.307	1.00	0.094	0.0072
Asv106383	Unclassified Ustilaginaceae	Plant pathogen	0.305	0.991	0.094	0.0072
Asv113385	Unclassified Fungus	-	0.303	0.869	0.106	0.027
Asv121159	*Adisciso* sp.	Plant pathogen	0.302	0.859	0.106	0.0479
Asv102174	Unclassified Ascomycota	-	0.288	0.88	0.094	0.0454

Asv97316	Unclassified Coniochaetaceae	Saprophyte, Plant pathogen, Symbiont	0.287	1.00	0.082	0.0129
Asv90510	*Saccharomyces* sp.	Saprophyte	0.287	1.00	0.082	0.0159
Asv132887	*Saccharyomyces eubayanus*	Saprophyte	0.287	1.00	0.082	0.0156
Asv106423	Unclassified Davidiellaceae	-	0.286	0.87	0.084	0.0308
Asv090063	*Coprinopsis sp.*	Saprophyte	0.272	0.899	0.082	0.0279
Asv108733	Unclassified Ustilaginaceae	Plant pathogen	0.27	0.884	0.082	0.0499
Asv90076	*Aspergillus* sp.	Saprophyte	0.266	0.858	0.082	0.0487
Asv97321	Unclassified Tremellales	Saprophyte, Fungal parasite	0.266	1.00	0.070	0.0306
Asv106384	Unclassified Ustilaginaceae	Plant pathogen	0.266	1.00	0.070	0.0293
Asv90165	*Aspergillus* sp.	Saprophyte	0.266	1.00	0.070	0.0304
Asv106399	*Malassezia* sp.	Saprophyte, Plant pathogen	0.266	1.00	0.070	0.0302
Asv101533	Unclassified Ascomycota	-	0.266	1.00	0.070	0.0267
Asv106427	*Malassezia* sp.	Saprophyte, Plant pathogen	0.266	1.00	0.070	0.0302
Asv90652	Unclassified Sordariomycetes	-	0.266	1.00	0.070	0.0268
Asv136051	*Penicillium* sp.	Saprophyte	0.266	1.00	0.070	0.0306
Asv102186	*Malassezia restricta*	Saprophyte, Plant pathogen	0.266	1.00	0.070	0.0272
Asv106565	Unclassified Davidiellaceae	-	0.266	1.00	0.070	0.0281
Asv106450	Unclassified Montagnulaceae	Saprophyte, Plant pathogen	0.266	1.00	0.070	0.0302
Asv97440	Unclassified Pleosporales	-	0.266	1.00	0.070	0.0275
Asv101559	Unclassified Trichosphaeriales	-	0.266	1.00	0.070	0.0297
