# Supplementary material for: Host-Environment Interplay Shapes Fungal Diversity in Mosquitoes
Source: mSphere. 2021 Sep 29;6(5):e00646-21. doi: 10.1128/mSphere.00646-21 (PMC8550294; doi:10.1128/mSphere.00646-21)
Supplement: TEXT S1 [file msphere.00646-21-t0001.pdf]

Tawidian *et al*. Supplementary Text

AMPLICON SEQUENCE VARIANT (ASV) ANALYSIS

To determine the contribution of sequence processing methodology on fungal community assembly in mosquito and water samples, we used amplicon sequence variants (ASVs) in addition to the OTU approach. A detailed description of the methodology can be found in the Materials and Methods section of the main manuscript. Here, we briefly describe the main findings of the ASV data analysis and compare its results with those obtained with the OTU dataset.

ASV methodology increases overall fungal richness and diversity. The ASV method increased fungal richness in the entire dataset by 2.3-fold as compared to the OTU method. This increase is expected as OTUs combine ASVs at the 97% sequence similarity, as is within the range of fold-increase of 1.3-4.1 reported in the two studies that compared these methodologies when analyzing fungal ITS sequence datasets (1, 2). Similarly, the ASV method increased the average Shannon's diversity index in the entire dataset by 1.1-fold as compared to the OTU method. As other studies have observed, the OTU and ASV observed and extrapolated richness and Shannon's diversity indices of individual samples were strongly correlated ($r_s = 0.9774$, $P < 0.0001$) (1, 2).

Fungal community composition is influenced by ITS sequence analysis method. The overall pattern of sequence distribution across phyla remained the same, with

Ascomycota, followed by Basidiomycota accounting combined for 90% of all reads (**Fig. S3A**). However, sequence processing methodology influenced fungal community composition with regards to the relative abundance of some specific taxa. This recapitulates the findings of other studies comparing OTU and ASV analyses for bacterial and/or fungal datasets (1–5). Below, we briefly summarize the taxa for which we observed the most prominent changes.

As observed in the OTU approach, the fungal orders Eurotiales, Pleosporales, and Hypocreales within the phylum Ascomycota were dominant, however shifted in their relative abundance as compared to the OTU method. In addition, Saccharomycetales was identified as an additional prominent order, claiming 10.7% of reads assigned to the phylum Ascomycota. Of the 447 ASVs assigned to Saccharomycetales, 316 were assigned to the yeast *Saccharomyces eubayanus*. This species was first isolated in 2011 from the bark of and soil surrounding Southern beech trees in Patagonia (6). Since then, this species has also been isolated from oak bark in Wisconsin, USA (7). In the Northern hemisphere, including the USA, *Saccharomyces sp.* are readily isolated from oak tree bark (8–10), which is the prominent tree genus in Manhattan, KS, and therefore is likely to be the environmental source of the *Saccharomyces* taxa in our dataset. Six ASVs assigned to the genus *Saccharomyces* were identified as gut indicator species via IndVal analysis (**Table S5**), and three were significantly associated with the gut tissue as compared to the carcass (**Table S6**). In contrast, no *Saccharomyces* taxa were indicator species of the carcass or associated with this tissue. This distribution strongly suggests that *Saccharomyces* is acquired by mosquito larvae through feeding on substrate in their larval habitat.

In contrast to the OTU dataset, the ASV analysis identified the order Malasseziales as dominant within the phylum Basidiomycota (33% of reads), and shared across all mosquito and water samples (**Fig. S3A**). The majority of the Malasseziales reads were assigned to two species, *Malassezia restricta* (48%) and *Malassezia globosa* (23%). Malassezia *sp*. are yeasts that are commonly found on healthy human skin and that are also associated with a variety of skin conditions (11). Based on culture-independent studies, *Malassezia* species are wide-spread and are found in ecologically diverse habitats (12). Some of these habitats are provided by other animals, and include the guts of marine organisms such as Japanese eel (13) and the larvae of the red rock lobster (14), as well as the exoskeleton of soil nematodes (15). Nines ASVs assigned to the genus *Malassezia* were identified as gut indicator species via IndVal analysis (**Table S5**), and seven were significantly associated with gut tissue as compared to the carcass (**Table S6**). In contrast, no *Malassezia* taxa were indicator species of the carcass or associated with this tissue. This distribution strongly suggests that like *Saccharomyces* yeast, *Malassezia* is acquired by mosquito larvae through feeding on substrate in their larval habitat.

ASV methodology confirms the larval breeding environment as a dominant factor shaping the fungal community assembly in mosquito larvae. We calculated the Bray-Curtis dissimilarities among all water, gut, and carcass ASV data followed by a PERMANOVA and MANOVA and visualized the distances by PCoA (**Fig. S3B**). The results paralleled those obtained using the OTU based approach and revealed significant differences in the fungal communities between samples from different sites

(**Figs. S2A and S3B**). In addition, we ran a MANOVA on the first three PCoA vectors. The MANOVA and univariate ANOVAs further confirmed that differences along all three PCoA axes were significant for site and the interaction of site and mosquito tissue type, and samples separated by tissue along the third axis (**Fig. S3C**).

Analysis of the ASV dataset confirms tissue-specific patterns of fungal diversity. Fungal communities of mosquito larval guts and carcasses, as assessed by OTU method differed in richness and diversity. Fungal OTU richness and diversity was consistently higher in mosquito gut samples than in the corresponding carcass samples. These overall patterns are reproduced when analyzing the ASV dataset. Alpha diversity differed between mosquitoes tissues as measured by observed (W = -3,212, < 0.0001) and extrapolative Chao1 (W = -3,164, P < 0.0001) ASV richness (**Fig. S3D**). However, in contrast to the OTU dataset, Shannon's diversity did not differ between guts and carcasses in the ASV dataset (W = -875, P < 0.0552). This is partially explained by an slightly higher average fold increase of Shannon's diversity in the carcass samples (1.15-fold) as compared to the gut samples (1.07-fold), as well as an only moderate correlation of OTU and ASV Shannon's diversity indices of individual carcass samples (r_s = 0.7688, P < 0.0001).

As observed with the OTU dataset, the pairwise Bray-Curtis dissimilarities derived from the ASV dataset continued to be higher between water and carcass samples than between water and gut samples **(Fig. S3E**), further confirming the tissue-specific patterns of fungal diversity between mosquito larval guts and carcasses.

SUPPLEMENTARY REFERENCES

1. Tipton L, Zahn GL, Darcy JL, Amend AS, Hynson NA. 2021. Hawaiian Fungal Amplicon Sequence Variants Reveal Otherwise Hidden Biogeography. Microb Ecol https://doi.org/10.1007/s00248-021-01730-x.

2. Glassman SI, Martiny JBH. 2018. Broadscale Ecological Patterns Are Robust to Use of Exact Sequence Variants versus Operational Taxonomic Units. mSphere 3.

3. Capunitan DC, Johnson O, Terrill RS, Hird SM. 2020. Evolutionary signal in the gut microbiomes of 74 bird species from Equatorial Guinea. Molecular Ecology 29:829–847.

4. Yakimovich KM, Engstrom CB, Quarmby LM. 2020. Alpine Snow Algae Microbiome Diversity in the Coast Range of British Columbia. Front Microbiol 11.

5. Moossavi S, Atakora F, Fehr K, Khafipour E. 2020. Biological observations in microbiota analysis are robust to the choice of 16S rRNA gene sequencing processing algorithm: case study on human milk microbiota. BMC Microbiology 20:290.

6. Libkind D, Hittinger CT, Valerio E, Goncalves C, Dover J, Johnston M, Goncalves P, Sampaio JP. 2011. Microbe domestication and the identification of the wild genetic stock of lager-brewing yeast. Proceedings of the National Academy of Sciences 108:14539–14544.

7. Peris D, Sylvester K, Libkind D, Gonçalves P, Sampaio JP, Alexander WG, Hittinger CT. 2014. Population structure and reticulate evolution of Saccharomyces eubayanus and its lager-brewing hybrids. Molecular Ecology 23:2031–2045.

8. Sniegowski PD, Dombrowski PG, Fingerman E. 2002. Saccharomyces cerevisiae and Saccharomyces paradoxus coexist in a natural woodland site in North America and display different levels of reproductive isolation from European conspecifics. FEMS Yeast Research 1:299–306.

9. Hittinger CT. 2013. Saccharomyces diversity and evolution: a budding model genus. Trends in Genetics 29:309–317.

10. Alsammar H, Delneri D. 2020. An update on the diversity, ecology and biogeography of the Saccharomyces genus. FEMS Yeast Res 20.

11. Saunders CW, Scheynius A, Heitman J. 2012. Malassezia Fungi Are Specialized to Live on Skin and Associated with Dandruff, Eczema, and Other Skin Diseases. PLOS Pathogens 8:e1002701.

12. Amend A. 2014. From Dandruff to Deep-Sea Vents: Malassezia-like Fungi Are Ecologically Hyper-diverse. PLOS Pathogens 10:e1004277.

13. Terahara T, Chow S, Kurogi H, Lee S-H, Tsukamoto K, Mochioka N, Tanaka H, Takeyama H. 2011. Efficiency of Peptide Nucleic Acid-Directed PCR Clamping and Its Application in the Investigation of Natural Diets of the Japanese Eel Leptocephali. PLOS ONE 6:e25715.

14. O’Rorke R, Lavery SD, Wang M, Nodder SD, Jeffs AG. 2014. Determining the diet of larvae of the red rock lobster (Jasus edwardsii) using high-throughput DNA sequencing techniques. Mar Biol 161:551–563.

15. Renker C, Alphei J, Buscot F. 2003. Soil nematodes associated with the mammal pathogenic fungal genus Malassezia (Basidiomycota: Ustilaginomycetes) in Central European forests. Biol Fertil Soils 37:70–72.
