# Supplementary material for: Host-Environment Interplay Shapes Fungal Diversity in Mosquitoes
Source: mSphere. 2021 Sep 29;6(5):e00646-21. doi: 10.1128/mSphere.00646-21 (PMC8550294; doi:10.1128/mSphere.00646-21)
Supplement: TABLE S6 [file msphere.00646-21-st006.pdf]

Table S6. Point-biserial correlation (r_{pb}) calculations of 48 ASVs with significant gut preference and seven ASVs with significant carcass preference across larval breeding sites.

ASVID	Taxa	Ecological guild	r_{pb}	p-value
**Gut**				
Asv90080	*Penicillium sp.*	Saprophyte	0.279	0.0004
Asv106383	Unclassified Ustilaginaceae	Plant pathogen	0.217	0.0043
Asv106391	Unclassified Montagnulaceae	Saprophyte, Plant pathogen, Fungal parasite	0.21	0.0043
Asv128755	*Malassezia restricta*	Saprophyte, Animal pathogen	0.209	0.001
Asv97302	Unclassified Montagnulaceae	Saprophyte, Plant pathogen	0.197	0.007
Asv90124	*Saccharomyces eubayanus*	Saprophyte	0.197	0.0081
Asv113293	*Aspergillus* sp.	Saprophyte	0.197	0.0029
Asv99680	Unclassified Sebacinales	-	0.192	0.0065
Asv97619	*Coprinellus xanthothrix*	Saprophyte	0.192	0.0011
Asv90198	*Malassezia globosa*	Saprophyte, Animal pathogen	0.189	0.0023
Asv090134	*Malassezia restricta*	Saprophyte, Animal pathogen	0.189	0.0023
Asv121166	*Thanatephorus cucumeris*	Plant pathogen	0.189	0.0111
Asv94461	Unclassified Ustilaginaceae	Plant pathogen	0.188	0.033
Asv100048	Unclassified Montagnulaceae	Saprophyte, Plant pathogen	0.186	0.004
Asv106380	Unclassified Montagnulaceae	Saprophyte, Plant pathogen	0.186	0.0043
Asv97357	*Gibberella intricans*	Plant pathogen	0.184	0.0051
Asv90938	*Paraconiothyrium* sp.	Saprophyte	0.182	0.0007
Asv97440	Unclassified Pleosporales	-	0.18	0.0315
Asv113385	Unclassified Fungus	-	0.177	0.014
Asv106399	*Malassezia* sp.	Saprophyte, Plant pathogen	0.176	0.0286
Asv121159	*Adisciso* sp.	Plant pathogen	0.175	0.0179
Asv97308	Unclassified Montagnulaceae	-	0.175	0.0132
Asv91643	*Trichoderma* sp.	Saprophyte	0.174	0.0071
Asv106925	Unclassified Eurotiomycetes	-	0.172	0.0154
Asv90063	*Coprinopsis sp.*	Saprophyte	0.17	0.0295
Asv106565	Unclassified Davidiellaceae	-	0.169	0.0268
Asv106450	Unclassified Montagnulaceae	Saprophyte, Plant pathogen, Fungal parasite	0.169	0.0286
Asv90589	Unclassified Trichosphaeriales	-	0.167	0.007
Asv97321	Unclassified Tremellales	Saprophyte, Fungal parasite	0.163	0.0275
Asv96534	*Cytospora* sp.	Saprophyte, Plant pathogen, Endophyte	0.163	0.0036
Asv90165	*Aspergillus* sp.	Saprophyte	0.162	0.0277
Asv99655	Unclassified Montagnulaceae	Saprophyte, Plant pathogen, Fungal parasite	0.161	0.0006
Asv90169	*Aspergillus brasiliensis*	Saprophyte	0.16	0.0283
Asv106427	*Malassezia* sp.	Saprophyte, Plant pathogen	0.158	0.0286
Asv106555	Unclassified Montagnulaceae	Saprophyte, Plant pathogen, Fungal parasite	0.157	0.0234
Asv128734	*Malassezia restricta*	Saprophyte, Animal pathogen	0.156	0.0073
Asv106384	*Paraconiothyrium* sp.	Saprophyte	0.156	0.0257
Asv136051	*Penicillium* sp.	Saprophyte	0.155	0.0298
Asv97316	Unclassified Coniochaetaceae	Saprophyte, Plant pathogen, Endophyte, Animal Pathogen, Lichen Parasite	0.151	0.0118
Asv132887	*Saccharyomyces eubayanus*	Saprophyte	0.145	0.0151

Asv101533	Unclassified Ascomycota	-	0.143	0.0279
Asv98754	*Aspergillus piperis*	Saprophyte	0.137	0.0497
Asv102186	*Malassezia restricta*	Saprophyte, Animal pathogen	0.134	0.0322
Asv121705	*Cordyceps bassiana*	Endophyte, Animal Pathogen, Fungal Parasite	0.127	0.0017
Asv101559	Unclassified Trichosphaeriales	-	0.125	0.0274
Asv91545	*Zopfiella marina*	Saprophyte	0.12	0.0001
Asv90652	Unclassified Sordariomycetes	-	0.117	0.0301
Asv90510	*Saccharomyces* sp.	Saprophyte	0.096	0.0148
**Carcass**				
Asv91544	Unclassified Eurotiomycetes	-	0.182	0.0109
Asv90101	Unclassified Davidiellaceae	-	0.170	0.0124
Asv92336	Unclassified Dothidiomycetes	-	0.153	0.0305
Asv97303	Unclassified Agaricomycetes	-	0.149	0.0204
Asv90954	*Cordyceps bassiana*	Endophyte, Animal Pathogen, Fungal Parasite	0.145	0.0298
Asv90362	Unclassified Agaricomycetes	-	0.143	0.0304
Asv92846	*Cladosporium velox*	Saprophyte, Plant Pathogen, Endophyte, Animal Pathogen, Lichen Parasite	0.123	0.0104
